# Deoxycholic Acid, a Secondary Bile Acid, Increases Cardiac Output and Blood Pressure in Rats

**DOI:** 10.3390/nu16010032

**Published:** 2023-12-21

**Authors:** Artur Nowiński, Dawid Chabowski, Joanna Giebułtowicz, Marta Aleksandrowicz, Marcin Ufnal

**Affiliations:** 1 Department of Experimental Physiology and Pathophysiology, Laboratory of Centre for Preclinical Research, Medical University of Warsaw, 02-106 Warsaw, Poland; chabowskidawid@gmail.com (D.C.); mufnal@wum.edu.pl (M.U.); 2 Department of Bioanalysis and Drugs Analysis, Faculty of Pharmacy, Medical University of Warsaw, 1 Banacha, 02-097 Warsaw, Poland; joanna.giebultowicz@wum.edu.pl; 3 Laboratory of Preclinical Research and Environmental Agents, Mossakowski Medical Research Institute, Polish Academy of Sciences, 02-106 Warsaw, Poland; maleksandrowicz@imdik.pan.pl

**Keywords:** deoxycholic acid, blood pressure, cardiac output, vasodilatation, bacterial metabolites

## Abstract

Background: Deoxycholic acid (DCA) is a secondary bile acid produced by gut bacteria. Elevated serum concentrations of DCA are observed in cardiovascular disease (CVD). We hypothesized that DCA might influence hemodynamic parameters in rats. Methods: The concentration of DCA in systemic blood was measured with liquid chromatography coupled with mass spectrometry. Arterial blood pressure (BP), heart rate (HR) and echocardiographic parameters were evaluated in anesthetized, male, 3–4-month-old Sprague–Dawley rats administered intravenously (IV) or intracerebroventricularly (ICV) with investigated compounds. Mesenteric artery (MA) reactivity was tested ex vivo. Results: The baseline plasma concentration of DCA was 0.24 ± 0.03 mg/L. The oral antibiotic treatment produced a large decrease in the concentration. Administered IV, the compound increased BP and HR in a dose-dependent manner. DCA also increased heart contractility and cardiac output. None of the tested compounds—prazosin (an alpha-blocker), propranolol (beta-adrenolytic), atropine (muscarinic receptor antagonist), glibenclamide (K-ATP inhibitor) or DY 268 (FXR antagonist), glycyrrhetinic acid (11HSD2 inhibitor)—significantly diminished the DCA-induced pressor effect. ICV infusion did not exert significant HR or BP changes. DCA relaxed MAs. Systemic vascular resistance did not change significantly. Conclusions: DCA elevates BP primarily by augmenting cardiac output. As a metabolite derived from gut bacteria, DCA potentially serves as a mediator in the interaction between the gut microbiota and the host’s circulatory system.

## 1. Introduction

An increasing body of evidence demonstrates a connection between CVD and gut bacteria metabolites [1]. Some studies provide evidence for associations between both gut dysbiosis and gut–blood barrier dysfunction with the pathophysiology of diseases, such as heart failure, metabolic syndrome, diabetes, and psychiatric disorders [2,3,4].

Bile acids (BAs) are a group of chemical compounds produced during cholesterol metabolism. Gut microbiota produces numerous vital nutrients for human homeostasis, such as vitamins K and B and short-chain fatty acids. Among these compounds are bile acids (BAs), produced during cholesterol metabolism. BAs are divided into two main groups: primary and secondary BAs. The formation process of the secondary BAs depends on gut microbiota, its composition, enzymatic activity, and the host’s general health [5]. One of the secondary BAs is deoxycholic acid (DCA), and bacterial enzymes are responsible for the modification of primary BAs and the production of DCA [6,7].

Researchers became interested in BAs in the context of the cardiovascular system in the 1860s when BAs were suggested to be responsible for bradycardia associated with jaundice [8]. In conditions that disrupt enterohepatic circulation or result in gut dysbiosis, there can be notable alterations in both the composition and concentrations of BAs in the blood and bile [5]. These changes, which can manifest as either increases or decreases in BA concentrations and alterations in the ratio of primary to secondary BAs, have been observed in a range of clinical conditions. Notable among these are non-alcoholic fatty liver disease, portal hypertension, bile stones, and cirrhosis [5,9,10,11].

CVD can lead to the impairment of systemic circulation and, subsequently, to the dysfunction of the gut–blood barrier [12]. BA composition and concentrations differ in patients with heart failure, especially the one affecting the right ventricle [13,14]. A high-fat diet is another factor assumed to be responsible for the increased permeability of the gut–blood barrier [15].

A few studies suggest a connection between DCA and diet composition, namely, a high-fat diet, which increases DCA levels in feces in humans and rats [16]. Likewise, a study shows a positive link between a high-fat and high-protein diet and secondary BA plasma concentration, including DCA [17]. On the other hand, other studies associated a high level of physical exercise, generally accepted as beneficial for one’s health (lowering the risk for developing high blood pressure, CV disease, or cancer), with lower levels of DCA and other BAs. The positive effect was observed both in humans and mice [18,19].

Under physiological conditions, a major portion of the BA is reabsorbed into the enterohepatic circulation, and a minor fraction is excreted with feces. A portion of this pool enters the systemic circulation, reaching various organs, including the brain [20]. BAs can cross the blood–brain barrier, and it has been suggested that BAs affect the central nervous system. BAs act as agonists for various receptors, including the FXR (farnesoid X receptor). The BAs brain tissue concentration depends on their overall concentration in the serum [21,22].

In individuals who have undergone cholecystectomy (CC), there is evidence of increased production of secondary BAs, a phenomenon documented in both experimental and clinical studies [11,23]. Additionally, with specific reference to deoxycholic acid (DCA), an increased incidence of certain types of cancer has been observed in patients who have undergone cholecystectomy [24]. It has been suggested that DCA possesses carcinogenic and mutagenic properties, and there is also evidence indicating that DCA itself can contribute to gut dysbiosis [25,26]. Taken together, these findings may elucidate the observed correlation between CC, dysbiosis, and cancer. Adding to this, another study highlighting the potential harmful effects of DCA in CC patients is an experimental research paper. It suggests a link between cholecystectomy, the accumulation of secondary BAs, dysbiosis, and the development of colitis [23]. Interestingly, an increased incidence of CVD, including conditions such as congestive heart failure and myocardial infarction, has been observed in subjects who have undergone CC [27].

There is little knowledge about DCA’s cardiovascular actions, especially in vivo. In the experimental setting, taurine-conjugated DCA (TDCA) action reduced spontaneous contractions of cultured rat cardiomyocytes in a time- and concentration-dependent manner [28]. TDCA is also reported to induce vasodilation of rodent aorta in the mechanism dependent on nitric oxide (NO) and muscarinic receptor activation [8]. Another study showed that TDCA causes vasorelaxation through interactions with voltage- and receptor-operated calcium channels [29]. However, the effects of DCA on the circulatory system in vivo are obscure.

This study aimed to clarify the hemodynamic effects of DCA in vivo in rats.

## 2. Materials and Methods

### 2.1. Compliance with Ethical Standards

The experiments were performed according to Directive 2010/63 EU on the protection of animals used for scientific purposes and approved by the I Local Bioethical Committee in Warsaw (permission no 474/2017).

### 2.2. Animals

Rats were housed in groups of 3 to 4 animals in polypropylene cages with 12 h light/dark cycle, temperature 22–23 °C, and humidity 45–55%, with unlimited access to standard laboratory diet and water.

Measurements were performed on 3–4-month-old male Sprague–Dawley rats, under general anesthesia with urethane at a dose of 1.5 g/kg body weight (BW). Rats were implanted with a polyurethane arterial catheter inserted through the femoral artery into the abdominal aorta and connected to the BP recording system. Heart rate (HR) was obtained from pulse wave. For IV treatment, a catheter was implanted into the femoral vein. All procedures were terminal.

### 2.3. The Effect of Antibiotic Treatment on DCA Plasma Concentration

In the pilot study, we tested the hypothesis that antibiotic treatment alters DCA plasma concentration. We treated the experimental group (*n* = 5) with neomycin and amoxicillin + clavulanic acid dissolved in drinking water (doses 50 mg/kg/day and 50 mg/kg/day + 12.5 mg/kg/day, respectively). The control group (*n* = 5) drank tap water. Blood samples were analyzed after 7 days of treatment.

### 2.4. Hemodynamic Effects of DCA Administered into the Cerebroventricular System

Anesthetized rats had an arterial catheter implanted, as described above. Next, the rats were implanted with a stainless-steel cannula (ID 0.7 mm × OD 0.9 mm), inserted into the lateral ventricle, and secured with dental cement. The intracerebroventricular (ICV) infusions were performed through a stainless-steel infusion tube inserted into the previously implanted cannula. The measurements began 75 min after the induction of anesthesia and 15 min after connecting the arterial catheter.

In separate series, rats were administered ICV (each *n* = 6) either a vehicle (10 µL of 1:1:3 ethyl alcohol:DMSO:0.9% saline solution), DCA 0.75 mmol/kg b.w., or DCA 0.375 mmol/kg b.w.

In separate series, rats were pretreated with DY 268 (50 nmol/kg) or glycyrrhetic acid (4 mg/kg) with DCA 0.375 mmol/kg b.w. or vehicle (control) infused ICV (each *n* = 6) 45 min after the pretreatment.

Hemodynamics were recorded for 20 min at baseline and 90 min after all ICV infusions. Cannula placement was controlled with methylene blue after decapitation.

### 2.5. Hemodynamic Effects of Sodium Deoxycholate Administered Intravenously

The measurements were started 60 min after the induction of anesthesia, and 15 min after connecting the arterial catheter. Hemodynamics were recorded for 20 min at baseline and for 90 min after the intravenous (IV) administration of either (1) a vehicle (0.5 mL of 0.9% saline), (2) DCA 4 mmol/kg, (3) DCA 12 mmol/kg, or (4) DCA 36 mmol/kg.

Due to significant hemodynamic response during IV administration of vehicle used in ICV infusions, for IV injections, we have used 0.9% saline as a vehicle and a solution of DOC in saline instead of pure DCA.

### 2.6. Blood Sampling

In separate series (each *n* =5), blood samples were taken at the moment of maximal hemodynamic response (5 min) after IV infusion for the dose of 4 mmol/kg BW and after normalization of BP (30 min). For the dose of 36 mmol/kg, the samples were taken 20 min after the infusion and at the end of the measurement (in accordance with earlier measurements). DCA concentrations were determined using liquid chromatography coupled with tandem mass spectrometry (LC-MS/MS)—the method description is in the supplemental material.

### 2.7. The Assessment of FXR and 11HSD2 Blockade on DCA Cardiovascular Actions

The precise mechanism of vascular and cardiovascular actions of BAs is unclear. We aimed to test the hypothesis that blockade of FXR, with DCA being one of its ligands, may affect DCA central and peripheral actions. The study by Zhang et al. shows possible involvement of this receptor in nitric-oxide-dependent vasorelaxation [30]. Another possible target of DCA is 11-hydroxysteroid dehydrogenase type 2 (11HSD2), determining possible pressor effects. Its activity is affected by DCA [31]. Therefore, we used DY 268—a selective FXR inhibitor and glycyrrhetinic acid—a compound exerting inhibitory actions on 11HSD2.

### 2.8. Mechanism of Action Assessment

In a separate series, the involvement of various receptors, with the use of DY 268 (150 nmol/kg), an antagonist of FXR receptor; 11-hydroxysteroid dehydrogenase type 2 (11HSD2) antagonist—glycyrrhetinic acid (GA) (20 mg/kg) [32]; α1 antagonist—prazosin (0.5 mg/kg) [33]; a non-selective β1 and β2 antagonist—propranolol (4 mg/kg) [34]; muscarinic receptors antagonist—atropine (1 mg/kg) [34]; glibenclamide (5 mg/kg)—non-selective K-ATP inhibitor [35], was tested.

After IV pretreatment with prazosin, propranolol, atropine, or glibenclamide (all in separate series), DCA 12 mmol/kg (*n* = 5) or 0.5 mL saline as control (*n* = 5) was injected.

Then, 45 min after pretreatment with DY 268 or GA (separate series), DCA 36 mmol/kg (*n* = 6) or 0.5 mL saline as control (*n* = 6) was injected. Hemodynamics were recorded for 20 min at baseline and at least 60 min after all infusions.

### 2.9. Ex vivo Reactivity Studies—Isolated Mesenteric Artery Studies

Isolation of third-order branches of rat mesenteric artery (MA) has been previously described in detail by Onyszkiewicz [36]. In brief, rats (*n* = 6) were anesthetized, and the MA branches (diameter not exceeding 350 µm) were dissected and placed in a petri dish filled with cold (4 °C, pH = 7.4) buffered physiological saline. DCA was administered in increasing concentration in the range 0.1–500 µM and the vascular effects were assessed after 15 min—a detailed description is in the supplemental material.

### 2.10. Echocardiography

Samsung HM70 (Seoul, South Korea) equipped with a linear probe 5–13 MHz was used. The rats were anesthetized and had arterial and venous catheters implanted. The examination was performed at the baseline (15 min after implantation) and 20 min after IV infusion of DCA 36 mmol/kg BW. Systemic vascular resistance (SVR) was calculated using a simplified equation (SVR = MAP/CO) and mean values for arterial blood pressure (MABP) and cardiac output (CO) registered 20 min after IV infusion of DCA 36 mmol/kg.

### 2.11. Chemicals

Urethane, deoxycholic acid, natrium deoxycholate, DMSO, glycyrrhetic acid, glibenclamide, and propranolol were obtained from Sigma-Aldrich (St. Louis, MO, USA). DY 268 and prazosin were obtained from Tocris (Bristol, UK). Atropine and neomycin were obtained from Polfa Warszawa (Warsaw, Poland). Amoxicillin + clavulanic acid was obtained from GlaxoSmithKline Export (Brentford, UK).

### 2.12. Data Analysis and Statistics

MABP and HR were calculated from the BP tracings by AcqKnowledge 5.0.3 software (Biopac Systems, Goleta, CA, USA). The Kolmogorov–Smirnov test was used to test the normality of the distribution. To evaluate MABP and HR response to the treatment, the average over 5 min baseline was compared with the averages over 5 min for the 90 min period after the treatment by means of one-way ANOVA for repeated measures. Differences between the series were evaluated by multivariate ANOVA, followed by Tukey’s post hoc test or *t*-test, when appropriate. A value of two-sided *p* < 0.05 was considered significant.

## 3. Results

### 3.1. The Effect of Antibiotic Treatment on DCA Plasma Concentration

DCA plasma concentrations differed (*p* < 0.01) between the antibiotic-treated and the control group. In the treated group, DCA concentration was below method sensitivity (LC-MS/MS) compared to the control group, 0.24 ± 0.03 mg/L.

### 3.2. Intravenous Infusions

**Figure 1:**
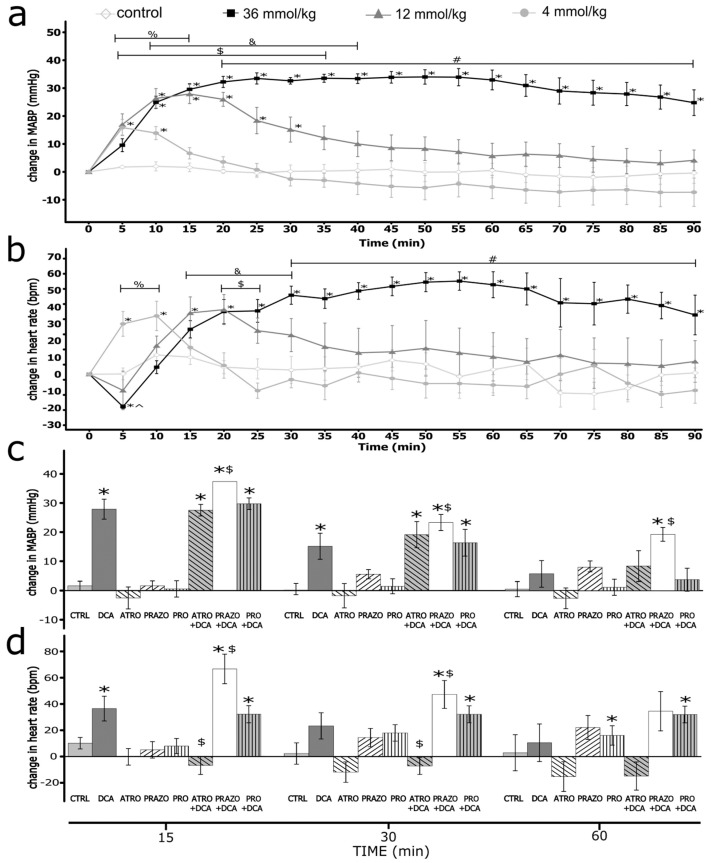
Changes in hemodynamic parameters in Sprague–Dawley rats after the intravenous administration (IV) of either a vehicle (0.9% NaCl) or deoxycholic acid (DCA) at a dose of 4, 12, and 36 mmol/kg. (**a**) Change in mean arterial blood pressure (ΔMABP, mmHg); * *p* < 0.05 vs. baseline, ^%^ *p* < 0.05: 4 mmol/kg DCA series vs. the vehicle, ^$^ *p* < 0.05: 12 mmol/kg DCA series vs. the vehicle, ^&^ *p* < 0.05: 12 mmol/kg vs. 4 mmol/kg DCA series, ^#^ *p* < 0.05: 36 mmol/kg DCA series vs. 4 mmol/kg, 12 mmol/kg DCA series and the vehicle. (**b**) Change in heart rate (ΔHR, beats/min); * *p* < 0.05 vs. baseline, ^%^ *p* < 0.05: 4 mmol/kg vs. 12 and 36 mmol/kg DCA series and the vehicle, ^&^ *p* < 0.05: 12 mmol/kg vs. 4 mmol/kg DCA series and the vehicle, ^$^ *p* < 0.05: 36 mmol/kg DCA series vs. the vehicle ^#^ *p* < 0.05: 36 mmol/kg DCA series vs. 4, 12 mmol/kg DCA series and the vehicle, ^ *p* < 0.05: 36 mmol/kg DCA vs. 4 mmol/kg series and the vehicle. (**c**) ΔMABP and (**d**) ΔHR after the intravenous infusions of DCA at a dose of 12 mmol/kg, or atropine (ATRO), prazosin (PRAZO), propranolol (PRO), the vehicle (CTRL), DCA after pretreatment with either atropine, prazosin, or propranolol (DCA + ATRO, DCA + PRAZO, DCA + PRO). * *p* < 0.05 vs. baseline, ^$^ *p* < 0.05 vs. DCA series. Means ± SE are presented.

Sodium deoxycholate produced an increase in blood pressure and heart rate (Figure 1).

After the infusion of DCA, we observed a significant increase in MABP. The magnitude and duration of the effect increased in a dose-dependent manner. The difference between the highest dose and control from the fifth minute was statistically significant. The intergroup difference was statistically significant (Figure 1).

Infusions of DCA produced significant changes in HR. For the dose of 36 mmol/kg, we observed a significant decrease in HR in the fifth minute, followed by a significant, long-lasting increase in HR. For the lower doses, we observed a dose-related increase in HR. The intergroup difference was statistically significant (Figure 1).

### 3.3. Atropine-, Prazosin-, and Propranolol-Induced Hemodynamic Changes

There were significant changes in hemodynamic parameters after pretreatment with prazosin, atropine, and propranolol (Table 1). Namely, we observed a significant decrease in MABP after pretreatment with prazosin. It promoted positive changes of MABP produced in the prazosin + DCA group for 10 min (Figure 1 and Figure S7), with lower absolute MABP values throughout the entire measurement (Figure S6).

Pretreatment with propranolol significantly decreased baseline MABP and did not influence changes in MABP produced by DCA (Figure 1, Figures S8 and S9). Other compounds did not significantly alter MABP (see the Supplemental Data).

After pretreatment with prazosin, HR decreased at baseline. DCA significantly increased HR throughout the measurement (Figure 1 and Figure S7). However, the absolute values were similar to IV infusion of DCA without pretreatment (Figure 1, Figures S6 and S7).

After pretreatment with propranolol, HR was decreased, and the decrease of HR caused by DCA in the first 5 min was blunted. Nevertheless, there were no significant differences during the rest of the measurements (Figure 1, Figures S8 and S9). 

After pretreatment with atropine, HR was increased, and the changes caused by DCA were blunted (Figure 1, Figures S4 and S5). Other compounds did not affect HR significantly (see the Supplemental Data).

### 3.4. DCA Plasma Concentration

For the highest dose (36 mmol/kg) administered IV, the concentration of DCA increased significantly at both timepoints (Table 2). For the lowest dose administered (4 mmol/kg), the concentration of DCA was elevated significantly after 5 min and comparable to baseline at 30 min (Table 2).

### 3.5. ICV Infusions

There were no significant differences in hemodynamic parameters between the series at baseline (Table 1).

Neither vehicle nor DCA or tested compounds produced significant changes in hemodynamic parameters (MABP, HR) in all ICV infusions (see the Supplemental Data).

### 3.6. Ex Vivo Reactivity Studies

**Figure 2:**
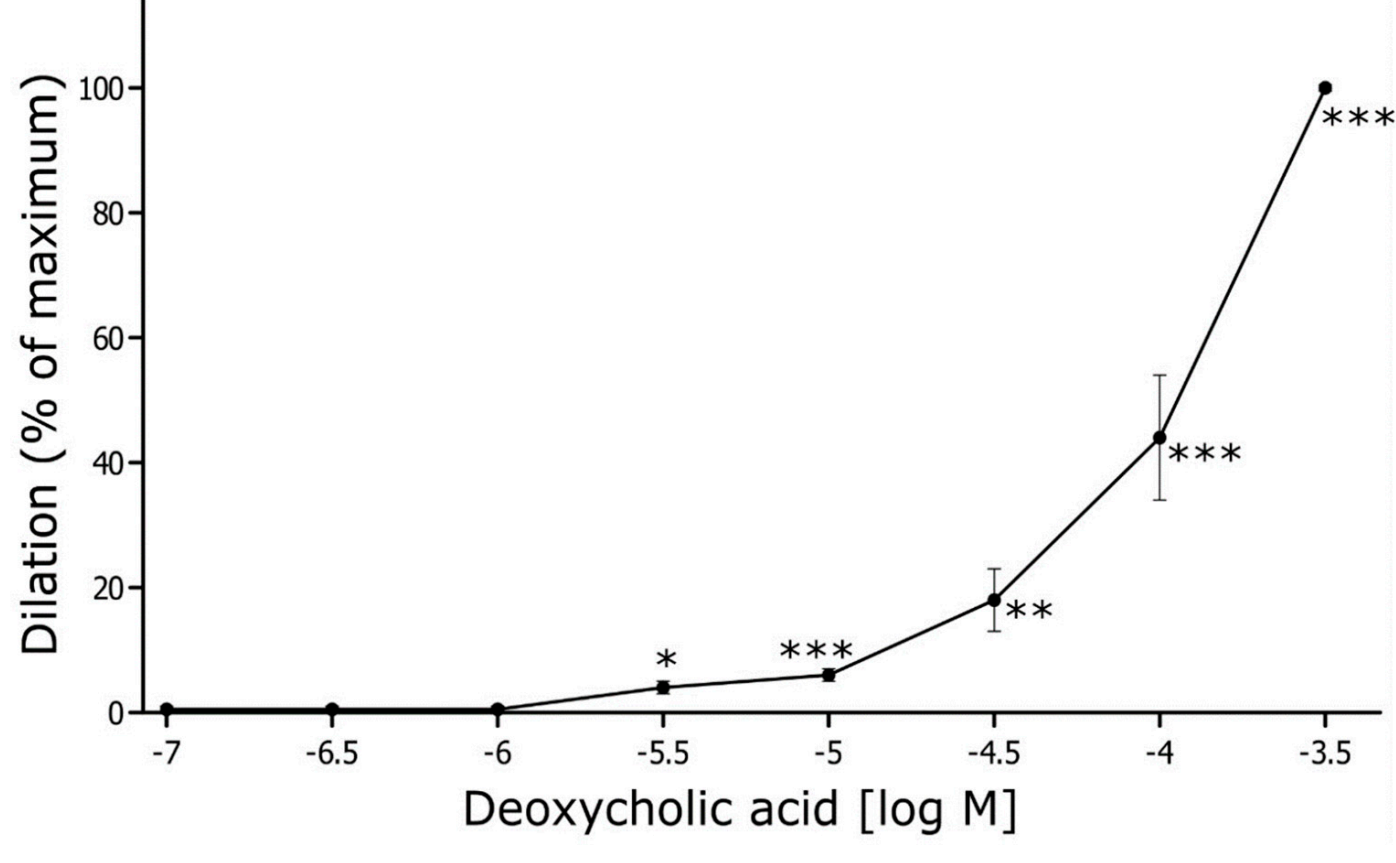
DCA-induced vascular relaxation in rat mesenteric artery. Mesenteric arteries were pre-contracted by phenylephrine (PE, 1 μM) and various concentrations of deoxycholic acid (from 0.1 µM up to 500 µM, *n* = 6) were applied extravascularly. Dilation is expressed as a percentage of maximum diameter. Means ± SE of *n* arteries are presented. * *p* < 0.05; ** *p* < 0.01; *** *p* < 0.001: significant vasorelaxation.

The mean initial internal diameter of the MAs was 315 ± 6 μm (*n* = 6). After preconstriction with phenylephrine (1 µM), the vessel diameter decreased on average by 57 ± 2%, reaching 133 ± 7 μm (*p* < 0.001). DCA, at the nearly physiological concentration (0.5 µM), did not change the diameter of the MAs. Nevertheless, DCA relaxed rat MA in a concentration-dependent manner (Figure 2). A significant relaxation of MA by 4 ± 1% was observed at the threshold concentration of 5 µM, and the maximal relaxation was reached at the concentration of 500 µM (Figure 2).

### 3.7. Echocardiography

Echocardiographic parameters are shown in Table 3. After IV infusion of DCA, significantly higher end-systolic intraventricular septum and end-systolic posterior wall thicknesses were observed. Pulmonary artery flow velocity was also significantly higher, and a trend towards a higher stroke volume (*p* = 0.06) was observed. There was a significant increase in calculated CO, but there were no differences in SVR.

## 4. Discussion

The new finding of our study is that IV infusion of deoxycholic acid increases BP by increasing CO. We observed an initial transient decrease in HR following IV administration of DCA. This decrease in HR appears to be a reflex response to the rapid increase in BP. The subsequent increase in HR suggests sympathoexcitation following the infusion. Furthermore, blunting of the HR response after atropine and propranolol supports the involvement of autonomic nervous regulation in the HR changes observed.

The effects of DCA were not mitigated by the various compounds we used, which were intended to evaluate specific vascular-dependent and cardiac-dependent mechanisms. Among these compounds were prazosin, a selective alpha1 blocker primarily acting as a vasodilator; propranolol, a non-selective beta-adrenolytic affecting both vessels and the heart; atropine, a non-selective antimuscarinic agent with vagolytic and vasodilating effects; and glibenclamide, a non-selective K-ATP channel inhibitor with potential for either vasodilation or vasoconstriction, depending on the site of action and involved mechanisms. Additionally, we used DY 268 and glycyrrhetinic acid (GA), with their mechanisms of action detailed earlier in the study.

Although some compounds (prazosin, propranolol, and atropine) affected HR changes during the measurements, the changes do not explain observed pressor effects after IV infusions. Alpha-, beta-, or M2-receptor-independent mechanisms may be involved, but their activation may differ in a DCA-concentration-related manner. No reduction in BP response after using propranolol or prazosin suggests mechanisms other than the ones probed to be responsible for the observed effects. Furthermore, echocardiographic parameters suggest improved contractility and higher flow. No increase in the EF and shortening fraction may be secondary to the tachycardiac response and, thus, different hemodynamics. Therefore, we hypothesize that elevated BP may be mainly the effect of increased cardiac output.

Ex vivo studies showed concentration-dependent vasorelaxation caused by DCA. However, at a physiological concentration (0.5 µM), DCA did not significantly change the arterial diameter. When extrapolating from the in vitro experiment results, concentrations measured after IV administration (for the highest dose 36 mmol/kg—13.7 µM at 15 min and 4.4 µM at 90 min) could produce arterial dilation of about 4–6%. However, in vivo experiments have shown no significant changes in calculated SVR, indicating that the systemic pressor mechanism outweighed the direct vasodilatory effect observed in ex vivo.

Despite conducting numerous experiments, we could not identify a direct mechanism of action for DCA on the heart and blood vessels. This suggests the possible involvement of unidentified receptors in the observed effects. Further research is warranted to elucidate these underlying mechanisms. The general results of our study may differ from previously published results because of the differences in methodology: longer measurements, different ways of administration, and dose titration.

In the context of previously reported ex vivo effects of DCA, the findings regarding its mechanisms of action are conflicting. Some studies suggest dependence on NO formation and muscarinic receptors [8,37]. Others [38] rule out NO, muscarinic receptors, and K^+^ channels. What remains common between those findings is the involvement of Ca^2+^ ions [29]. Ca^2+^ ions are involved in the function of large-conductance Ca^2+^-activated K^+^ channels (BK calcium channels), which affect the membrane potential. This effect may be mediated by some BAs, including DCA, as described by Dopico and Bukiya [39]. Alternatively, DCA has been shown to increase intracellular Ca^2+^ concentration in vascular endothelial cells in in vitro studies [29,37]. We cannot rule out the notion that DCA action on cardiomyocytes may lead to a similar effect—an increased cytoplasmic Ca^2+^ concentration—a mechanism that may underly a positive inotropic effect [40].

Finally, we did not observe any significant hemodynamic changes after ICV infusions, suggesting DCA does not produce an acute response in the central nervous system.

It must be stressed that various BAs naturally occur as a mixture, so their net effect on hemodynamics may differ from the action of a single metabolite. Furthermore, we investigated the acute effects in general anesthesia. Anesthetics can significantly influence the regulation of the circulatory system. Therefore, further studies assessing chronic hemodynamic changes in freely moving animals are needed.

Altogether, our findings suggest that DCA is one of the gut-microbiota-produced agents that may contribute to the effect of gut bacteria on the host’s circulatory system homeostasis.

## 5. Conclusions

DCA elevates BP primarily by augmenting cardiac output. As a metabolite derived from gut bacteria, DCA potentially serves as a mediator in the interaction between the gut microbiota and the host’s circulatory system. The mechanism of action seems to be independent of α1, β1, β2, FXR receptors, and K-ATP channels. Further studies are needed to evaluate the mechanism involved in the pressor effect of DCA.

## Figures and Tables

**Table 1:** Baseline mean arterial blood pressure (MABP, mmHg) and heart rate (HR, beats/min).

Series	MABP	HR
Intravenous Infusions		
Vehicle	90.4 ± 3.0	331.3 ± 14.4
DOC 4 mmol/kg	86.0 ± 4.5	312.2 ± 17.2
DOC 12 mmol/kg	87.5 ± 4.0	324.1 ± 9.9
DOC 36 mmol/kg	89.1 ± 3.3	339.6 ± 9.8
Atropine	87.9 ± 1.3	393.0 ± 11.9 *
Atropine + DOC	84.0 ± 1.9	392.0 ± 11.9 *
Prazosin	55.3 ± 1.0 *	277.4 ± 12.9 *
Prazosin + DOC	59.1 ± 1.7 *	286.5 ± 10.4 *
Propranolol	77.7 ± 3.4 *	243.7 ± 16.7 *
Propranolol + DOC	78.2 ± 3.0 *	251.0 ± 10.1 *
DY 268	90.0 ± 3.5	333.4 ± 20.1
DY 268 + DOC	90.6 ± 2.6	330.3 ± 14.0
GA	89.8 ± 3.4	330.8 ± 14.3
GA + DOC	88.6 ± 3.4	327.7 ± 8.3
Intracerebroventricular Infusions		
Vehicle	89.7 ± 3.7	354.7 ± 19.4
DOC 0.375 mmol/kg	89.2 ± 3.5	349.2 ± 14.4
DOC 0.75 mmol/kg	89.5 ± 3.4	353.1 ± 14.2
DY 268	87.9 ± 2.6	367.7 ± 5.9
DY 268 + DOC	85.9 ± 4.0	365.9 ± 13.8
GA	86.1 ± 4.9	367.8 ± 14.1
GA + DOC	84.9 ± 2.8	370.2 ± 14.4

Means ± SE are presented. * *p* < 0.05 for differences between the series.

**Table 2:** Comparison of DCA plasma concentration at different timepoints after IV administration (36 and 4 mmol/kg bw).

Timepoints	Deoxycholic Acid Concentration ± SE (mg/L)	*p* vs. Baseline	*p* vs. Timepoints
Dose: 36 mmol/kg bw			
Baseline	0.215 ± 0.075	-	-
20 min	5.630 ± 0.571	<0.001	0.002
90 min	1.801 ± 0.171	0.003	0.002
Dose: 4 mmol/kg bw			
Baseline	0.206 ± 0.068	-	-
5 min	0.844 ± 0.177	0.027	0.028
30 min	0.344 ± 0.048	0.143	0.028

Plasma concentrations at baseline and described timepoints after intravenous infusion of DCA 36 or 4 mmol/kg bw. Means ± SE are presented. Dependent *t*-test was used.

**Table 3:** Echocardiographic parameters at different timepoints after the of infusion of DCA. Timepoints: baseline (T1) and 20 min after the end of infusion (T2). Mean values ± SE are presented. * *p* < 0.05 between timepoints. Systemic vascular resistance (SVR) is calculated using equation described earlier in the manuscript.

Parameter	T1	T2
IVSD (cm)	0.192 ± 0.006	0.194 ± 0.003
LVDD	0.571 ± 0.034	0.571 ± 0.019
PWD	0.208 ± 0.009	0.214 ± 0.009
IVSS	0.265 ± 0.007	0.291 ± 0.010 *
LVDS	0.318 ± 0.015	0.325 ± 0.012
PWS	0.305 ± 0.009	0.327 ± 0.019 *
EF %	79.57 ± 2.158	79.71 ± 3.121
FS %	43.14 ± 2.126	44.14 ± 3.752
LV EDV	0.472 ± 0.064	0.457 ± 0.046
LV ESV	0.090 ± 0.010	0.081 ± 0.010
Stroke volume (mL)	0.352 ± 0.039	0.391 ± 0.033
AO (cm)	0.365 ± 0.010	0.375 ± 0.009
LA (cm)	0.440 ± 0.007	0.432 ± 0.019
LA/AO	1.202 ± 0.036	1.10 ± 0.033
HR (bpm)	320 ± 9.419	388.8 ± 10.795 *
PA Vmax (m/s)	0.638 ± 0.051	0.737 ± 0.049 *
CO (mL/min)	112.138 ± 11.561	151.03 ± 10.647 *
SVR (PRU)	0.791 ± 0.123	0.803 ± 0.105

Abbreviations, in order of appearance: IVSD—interventricular septum diastolic thickness, LVDD—left ventricular end-diastolic diameter, PWD—posterior wall diastolic thickness, IVSS—interventricular septum systolic thickness, LVDS—left ventricular end-systolic diameter, PWS—posterior wall systolic thickness, EF—ejection fraction, FS—shortening fraction, LV EDV—left ventricular end-diastolic volume, LV ESV—left ventricular end-systolic volume, AO—aortic diameter, LA—left atrial diameter, HR—heart rate, PA Vmax—pulmonary artery maximum systolic velocity, CO—cardiac output, SVR—systemic vascular resistance.

## Data Availability

All available data are presented in the manuscript or in the supplementary material.

## Supplementary Materials

The following supporting information can be downloaded at: https://www.mdpi.com/article/10.3390/nu16010032/s1, Figure S1: Changes in hemodynamic parameters in Sprague-Dawley rats after the intravenous administration (IV) of either a vehicle (0.9% NaCl) or deoxycholic acid (DCA) at doses of 4, 12, and 36 mmol/kg. Figure S2: Mean arterial blood pressure (MABP) in Sprague-Dawley rats after the intravenous administration (IV) of either a vehicle (0.9% NaCl) or DCA at doses of 4, 12, and 36 mmol/kg. Figure S3: Mean heart rate (HR) in Sprague-Dawley rats after the intravenous administration (IV) of either a vehicle (0.9% NaCl) or deoxycholic acid (DCA) at doses of 4, 12, and 36 mmol/kg. Figure S4: Hemodynamic parameters in Sprague-Dawley rats after the intravenous administration (IV) of deoxycholic acid (DCA) at a dose of 12 mmol/kg without pretreatment or after pretreatment with atropine: DCA at a dose 12 mmol/kg (atropine + DCA) or the vehicle (control). Figure S5: Changes in hemodynamic parameters in Sprague-Dawley rats after the intravenous administration (IV) of deoxycholic acid (DCA) at a dose of 12 mmol/kg without pretreatment or after pretreatment with atropine: DCA at a dose of 12 mmol/kg (atropine + DCA) or the vehicle (control). Figure S6: Hemodynamic parameters in Sprague-Dawley rats after the intravenous administration (IV) of deoxycholic acid (DCA) at a dose of 12 mmol/kg without pretreatment or after pretreatment with prazosin: DCA at a dose of 12 mmol/kg (prazosin + DCA) or the vehicle (control). Figure S7: Changes in hemodynamic parameters in Sprague-Dawley rats after the intravenous administration (IV) of deoxycholic acid (DCA) at a dose of 12 mmol/kg without pretreatment or after pretreatment with prazosin: DCA at a dose of 12 mmol/kg (prazosin + DCA) or the vehicle (control). Figure S8: Hemodynamic parameters in Sprague-Dawley rats after the intravenous administration (IV) of deoxycholic acid (DCA) at a dose of 12 mmol/kg without pretreatment or after pretreatment with propranolol: DCA at a dose of 12 mmol/kg (propranolol + DCA) or the vehicle (control). Figure S9: Changes in hemodynamic parameters in Sprague-Dawley rats after the intravenous administration (IV) of deoxycholic acid (DCA) at a dose of 12 mmol/kg without pretreatment or after pretreatment with propranolol: DCA at a dose of 12 mmol/kg (propranolol + DCA) or the vehicle (control). Figure S10: Hemodynamic parameters in Sprague-Dawley rats after the intravenous administration (IV) of deoxycholic acid (DCA) at a dose of 36 mmol/kg or the vehicle (control) without pretreatment or after pretreatment with DY 268: DCA at a dose of 36 mmol/kg (DY + DCA) or the vehicle (DY group). Figure S11: Changes in hemodynamic parameters in Sprague-Dawley rats after the intravenous administration (IV) of deoxycholic acid (DCA) at a dose of 36 mmol/kg or the vehicle (control) without pretreatment or after pretreatment with DY 268: DCA at a dose of 36 mmol/kg (DY + DCA) or the vehicle (DY group). Figure S12: Hemodynamic parameters in Sprague-Dawley rats after the intravenous administration (IV) of deoxycholic acid (DCA) at a dose of 36 mmol/kg or the vehicle (control) without pretreatment or after pretreatment with glycyrrhetinic acid: DCA at a dose of 36 mmol/kg (GA + DCA) or the vehicle (GA group). Figure S13: Changes in hemodynamic parameters in Sprague-Dawley rats after the intravenous administration (IV) of deoxycholic acid (DCA) at a dose of 36 mmol/kg or the vehicle (control) without pretreatment or after pretreatment with glycyrrhetinic acid: DCA at a dose of 36 mmol/kg (GA + DCA) or the vehicle (GA group). Figure S14: Hemodynamic parameters in Sprague-Dawley rats after the intracerebroventricular (ICV) administration of either the vehicle or deoxycholic acid (DCA) at a dose of 0.375 or 0.75 mmol/kg. Figure S15: Changes in hemodynamic parameters in Sprague-Dawley rats after the intracerebroventricular (ICV) administration of either the vehicle or deoxycholic acid (DCA) at a dose of 0.375 or 0.75 mmol/kg. Figure S16: Hemodynamic parameters in Sprague-Dawley rats after the intracerebroventricular (ICV) administration of either the vehicle or deoxycholic acid (DCA) at a dose of 0.75 mmol/kg after pretreatment with DY 268. Figure S17: Changes in hemodynamic parameters in Sprague-Dawley rats after the intracerebroventricular (ICV) administration of either the vehicle or deoxycholic acid (DCA) at a dose of 0.75 mmol/kg after pretreatment with DY 268. Figure S18: Hemodynamic parameters in Sprague-Dawley rats after the intracerebroventricular (ICV) administration of either the vehicle or deoxycholic acid (DCA) at a dose of 0.75 mmol/kg after pretreatment with glycyrrhetinic acid. Figure S19: Changes in hemodynamic parameters in Sprague-Dawley rats after the intracerebroventricular (ICV) administration of either the vehicle or deoxycholic acid (DCA) at a dose of 0.75 mmol/kg after pretreatment with glycyrrhetinic acid.
