# Supplementary material for: Deoxycholic Acid, a Secondary Bile Acid, Increases Cardiac Output and Blood Pressure in Rats
Source: Nutrients. 2023 Dec 21;16(1):32. doi: 10.3390/nu16010032 (PMC10781055; doi:10.3390/nu16010032)
Supplement: Supplementary file 1 [file nutrients-16-00032-s001.zip › nutrients-2744714-supplementary.pdf]

Title: Deoxycholic acid, a secondary bile acid, increases cardiac output and blood pressure in rats.

INTRAVENOUS ADMINISTRATION

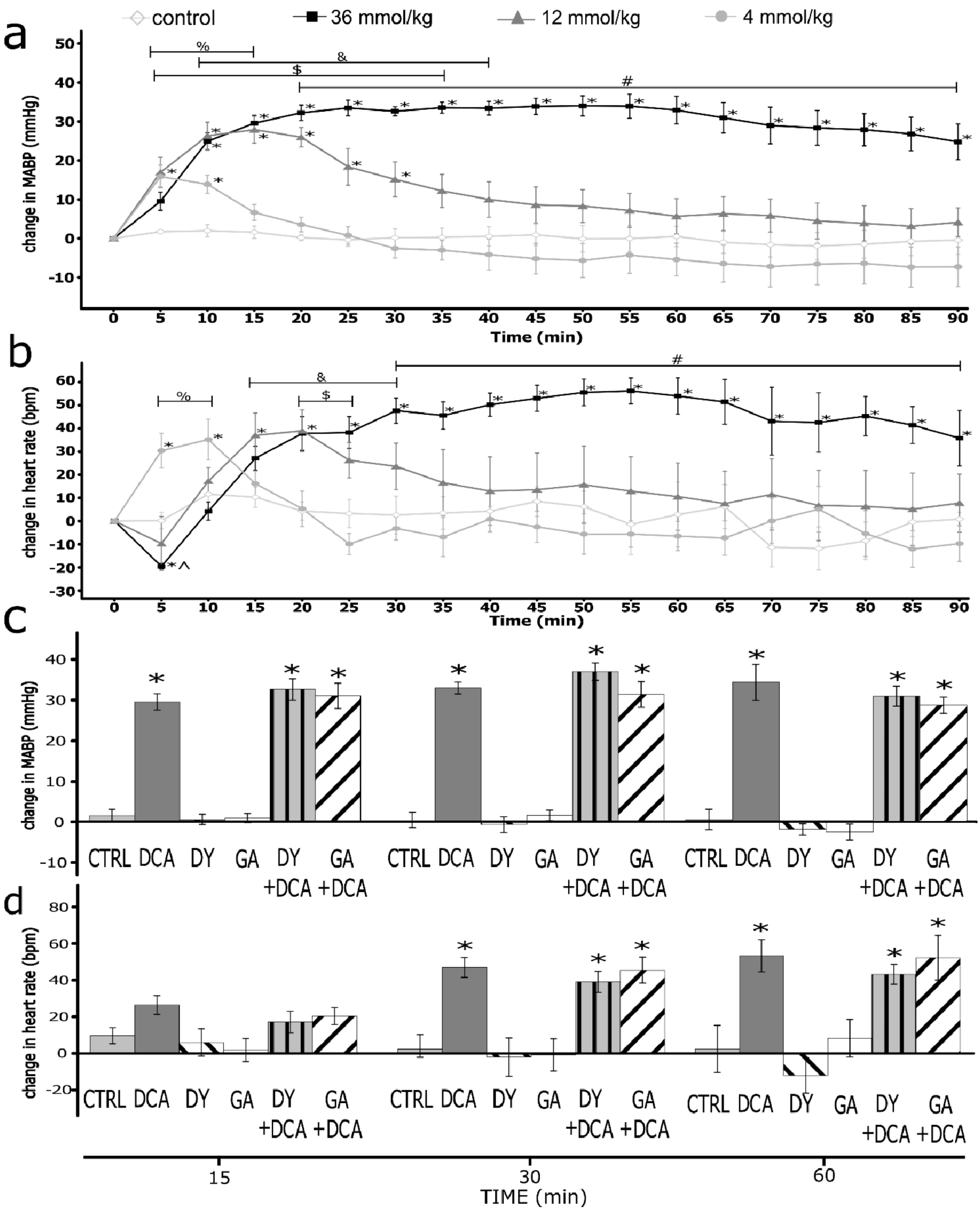

Figure S1

Changes in hemodynamic parameters in Sprague-Dawley rats after the intravenous administration (IV) of either a vehicle (0.9% NaCl) or deoxycholic acid (DCA) at doses of 4, 12, and 36 mmol/kg

a) mean arterial blood pressure (ΔMABP, mmHg); ***p**<0.05 vs. baseline, $^{\%}p<0.05$: 4 mmol/kg DCA series vs. the vehicle, $^{\$}p<0.05$: 12 mmol/kg DCA series vs. the vehicle, $^{\&}$p<0.05: 12 mmol/kg vs. 4 mmol/kg DCA series, $^{\#}$p<0.05: 36 mmol/kg DCA series vs. 4, 12 DCA series and the vehicle.

b) heart rate (ΔHR, beats/min); ***p**<0.05 vs. baseline, $^{\%}p<0.05$: 4 mmol/kg vs. 12 and 36 mmol/kg DCA series and the vehicle, $^{\&}$p<0.05: 12 mmol/kg vs. 4 mmol/kg DCA series and the vehicle, $^{\$}p<0.05$: 36 mmol/kg DCA series vs. the vehicle, $^{\#}$p<0.05: 36 mmol/kg DCA series vs. 4, 12 mmol/kg DCA series and the vehicle, ^p<0.05: 36 mmol/kg DCA vs. 4 mmol/kg series and the vehicle.

c) and **d)** ΔMABP and ΔMHR after the intravenous infusions of deoxycholic acid (DCA) at a dose of 36 mmol/kg (DCA), or DY 268 (DY), or glycyrrhetic acid (GA) or the vehicle (CTRL), or DCA after pretreatment with either DY 268 or glycyrrhetic acid (DCA+DY, DCA+GA). ***p**<0.05 vs. baseline.

Means ± SE are presented

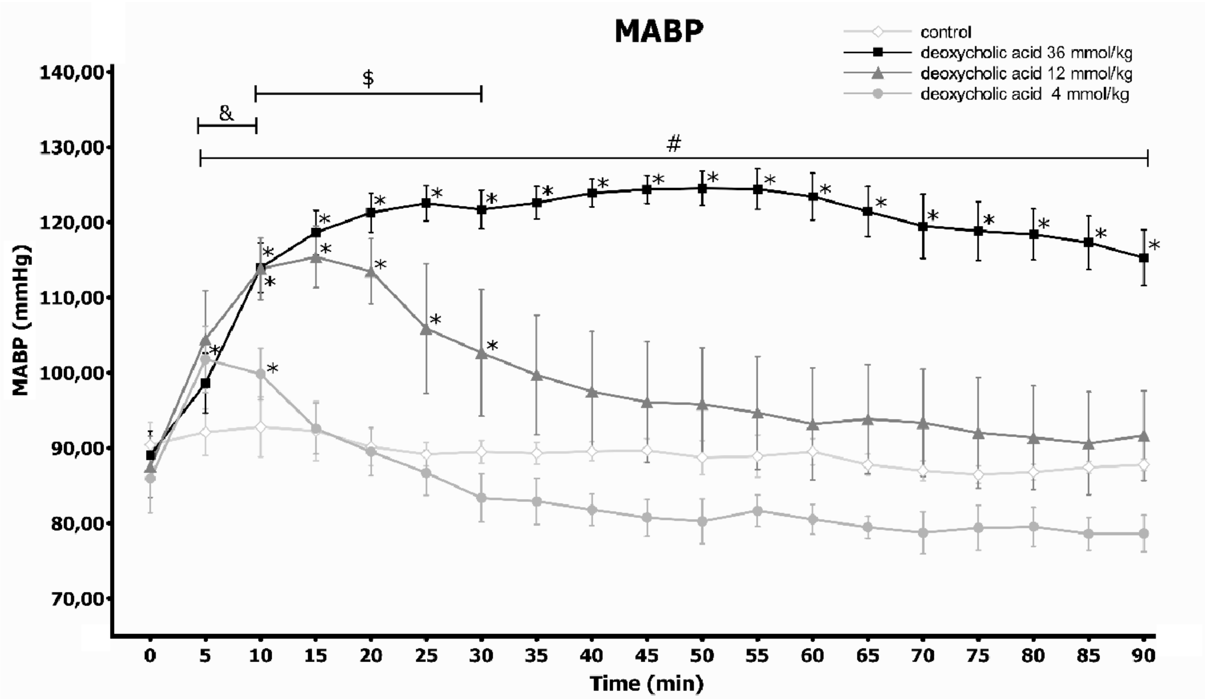

Figure S2

Mean arterial blood pressure (MABP) in Sprague-Dawley rats after the intravenous administration (IV) of either a vehicle (0.9% NaCl) or DCA at doses of 4, 12, and 36 mmol/kg; *****$p<0.05$ vs. baseline, $^{\%}p<0.05$: 4 mmol/kg DCA series vs. the vehicle, $^{\$}p<0.05$: 12 mmol/kg DCA series vs. the vehicle, $^{\&}p<0.05$: 4 mmol/kg DCA series vs. vehicle. Means ± SE are presented

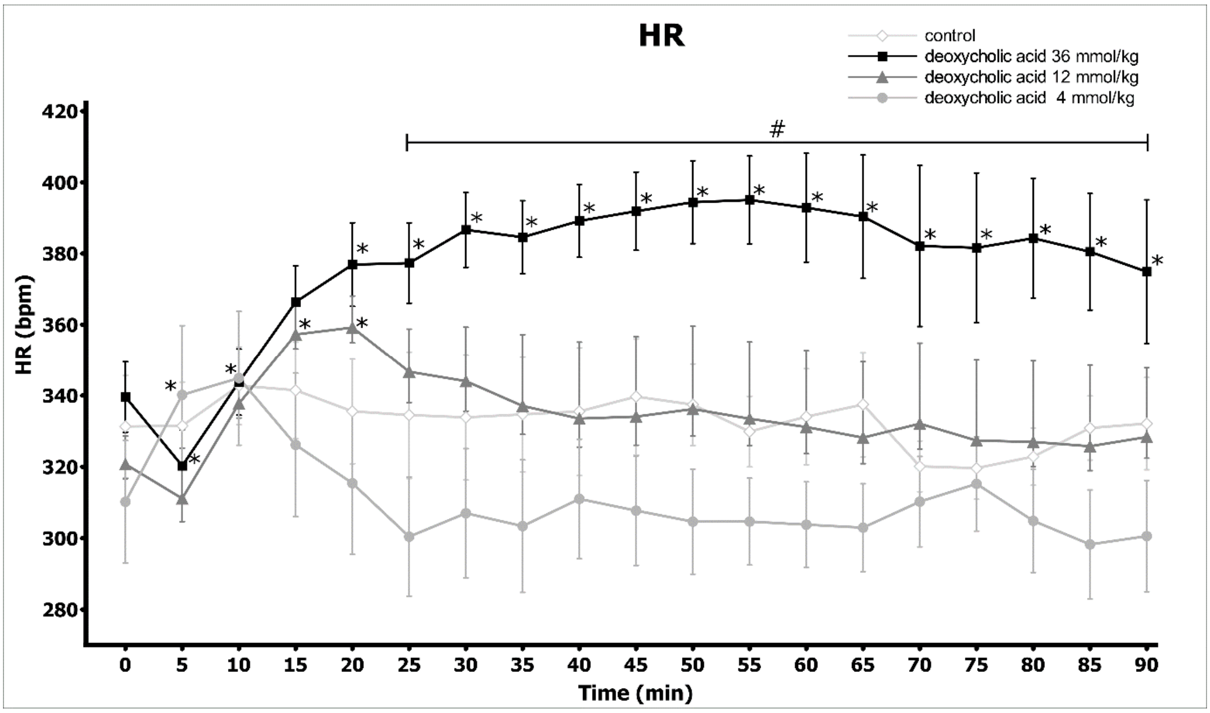

Figure S3

Mean heart rate (HR) in Sprague-Dawley rats after the intravenous administration (IV) of either a vehicle (0.9% NaCl) or deoxycholic acid (DCA) at doses of 4, 12, and 36 mmol/kg; ***p**<0.05 vs. baseline, #p<0.05: 36 mmol/kg DCA vs. the vehicle. Means ± SE are presented

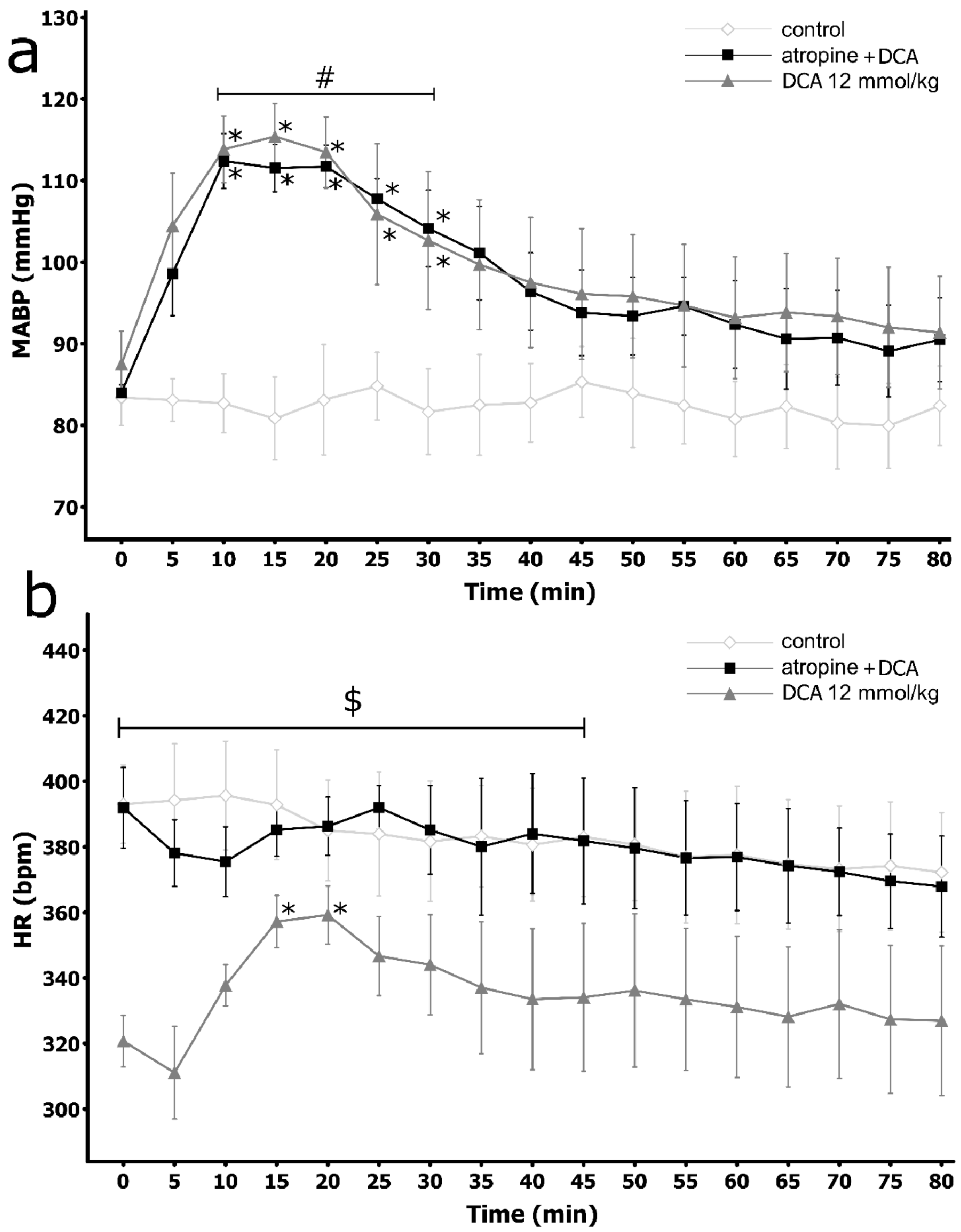

Figure S4

Hemodynamic parameters in Sprague-Dawley rats after the intravenous administration (IV) of deoxycholic acid (DCA) at a dose of 12mmol/kg without pretreatment or after pretreatment with atropine: DCA at a dose 12 mmol/kg (atropine+DCA) or the vehicle (control): **a.** Mean arterial blood pressure (MABP, mmHg); **b.** Heart rate (HR, bpm); ***p<0.05 vs. baseline, $^{\#}p$<0.05: atropine + DCA vs. control, $^{\$}p$<0.05: atropine + DCA vs. DCA. Means ± SE are presented

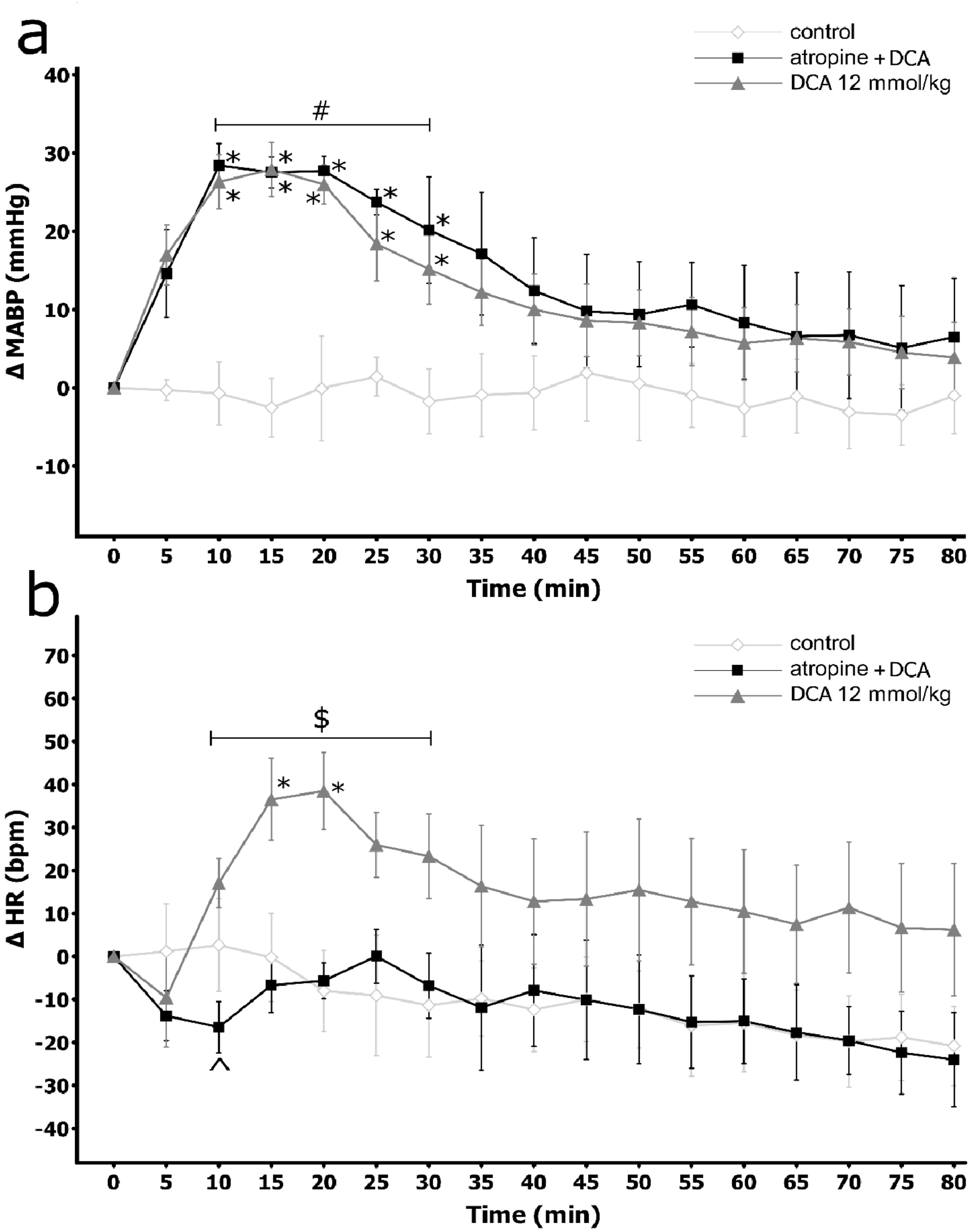

Figure S5

Changes in hemodynamic parameters in Sprague-Dawley rats after the intravenous administration (IV) of deoxycholic acid (DCA) at a dose of 12mmol/kg without pretreatment or after pretreatment with atropine: DCA at a dose of 12 mmol/kg (atropine+DCA) or the vehicle (control): **a.** ΔMABP (mmHg), **b.** ΔHR (bpm); ***p<0.05 vs. baseline, $^{\#}p$<0.05: atropine + DCA vs. control, $^{\$}p$<0.05: atropine + DCA vs. DCA, $^{\wedge}p$<0.05: atropine + DCA vs. atropine. Means ± SE are presented

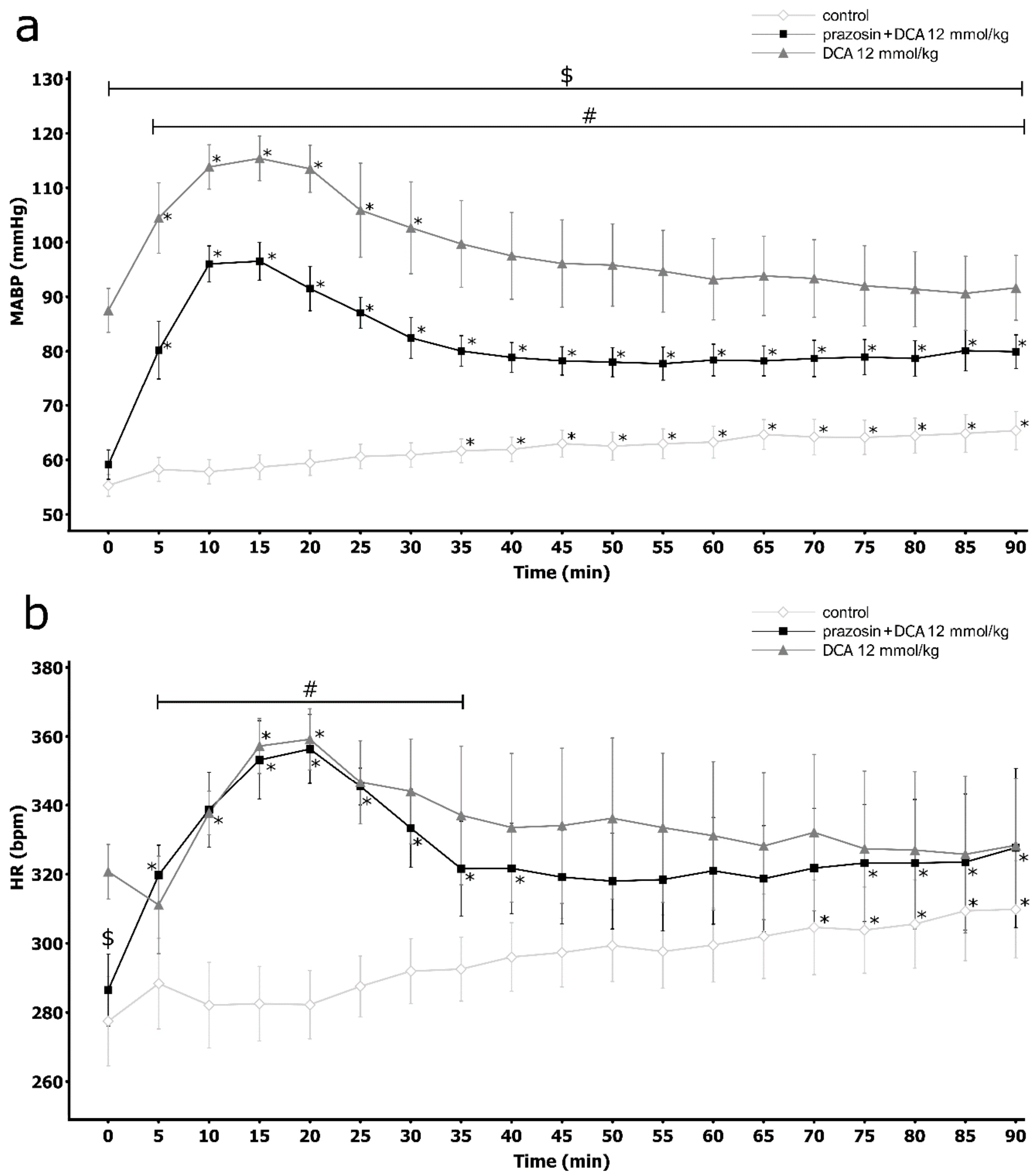

Figure S6

Hemodynamic parameters in Sprague-Dawley rats after the intravenous administration (IV) of deoxycholic acid (DCA) at a dose of 12mmol/kg without pretreatment or after pretreatment with prazosin: DCA at a dose of 12 mmol/kg (prazosin+DCA) or the vehicle (control); **a.** Mean arterial blood pressure (MABP, mmHg), **b.** Heart rate (HR, bpm); ***p<0.05 vs baseline, $^{\#}p$<0.05: prazosin + DCA vs. control $^{\$}p$<0.05: prazosin + DCA vs. DCA. Means ± SE are presented

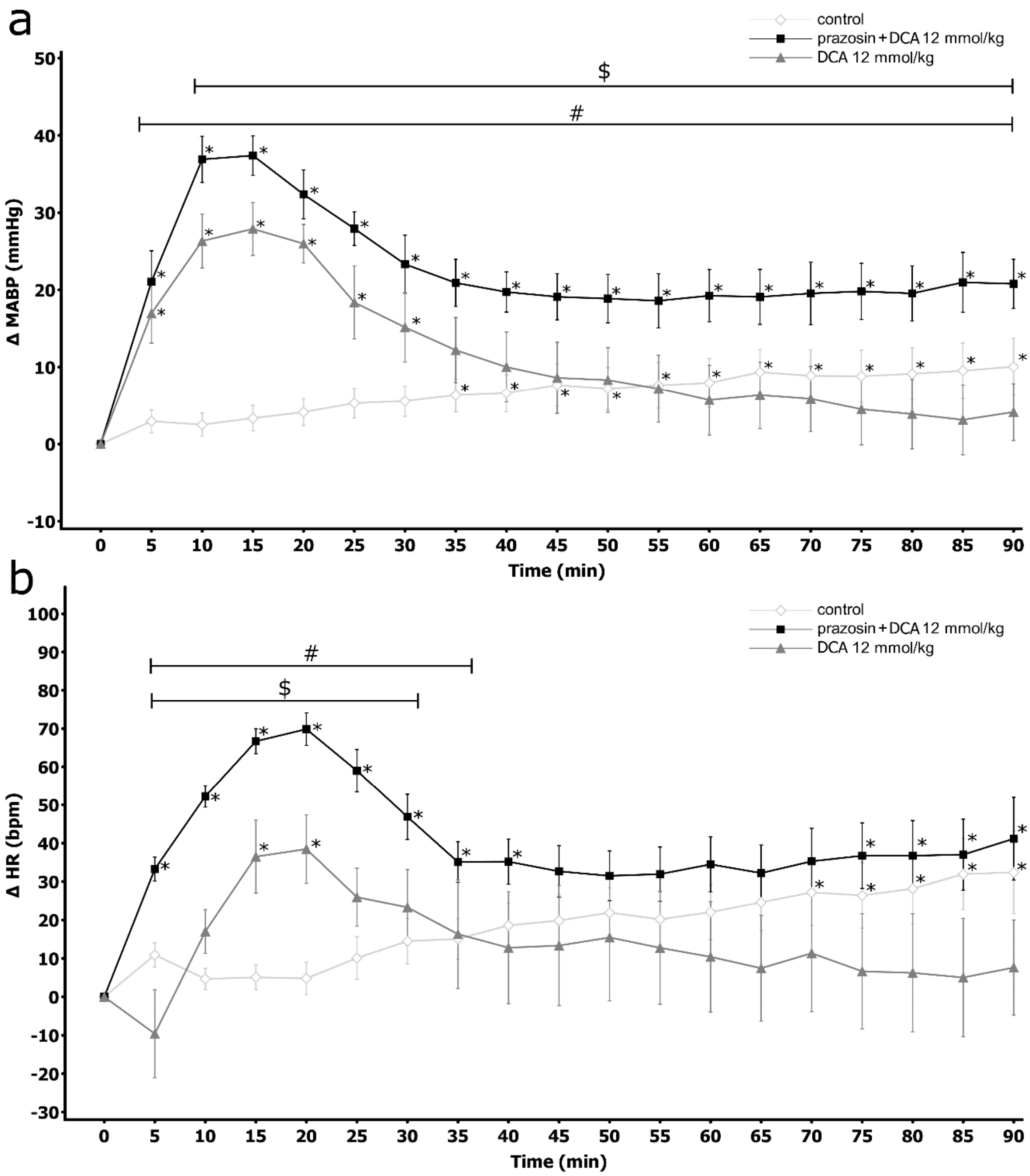

Figure S7

Changes in hemodynamic parameters in Sprague-Dawley rats after the intravenous administration (IV) of deoxycholic acid (DCA) at a dose of 12mmol/kg without pretreatment or after pretreatment with prazosin: DCA at a dose of 12 mmol/kg (prazosin+DCA) or the vehicle (control): **a.** ΔMABP (mmHg), **b.** ΔHR (bpm); *****$p<0.05$ vs. baseline, # $p<0.05$: prazosin + DCA vs. control $ $p<0.05$: prazosin + DCA vs. DCA. Means ± SE are presented

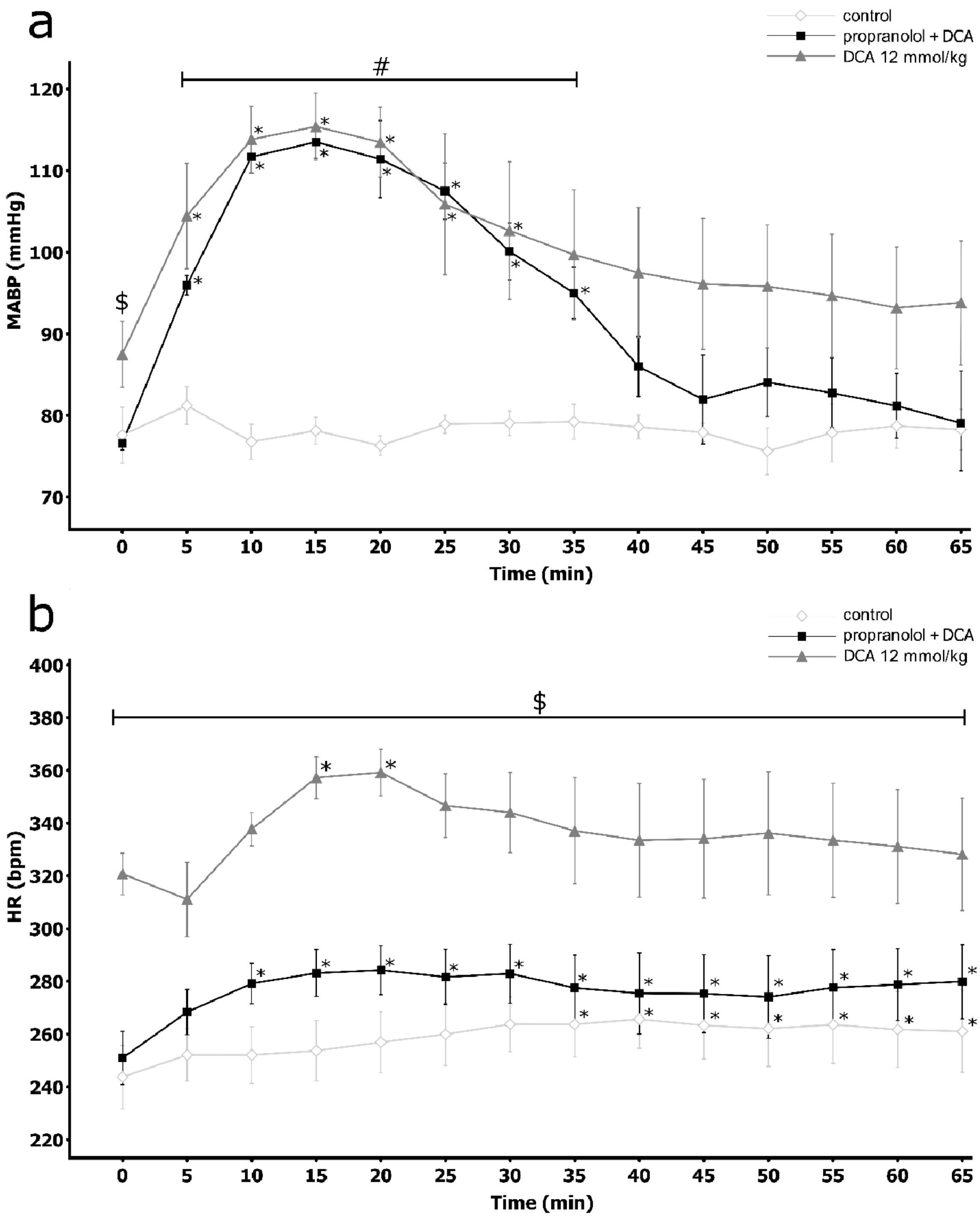

Figure S8

Hemodynamic parameters in Sprague-Dawley rats after the intravenous administration (IV) of deoxycholic acid (DCA) at a dose of 12mmol/kg without pretreatment or after pretreatment with propranolol: DCA at a dose of 12 mmol/kg (propranolol+DCA) or the vehicle (control): **a.** Mean arterial blood pressure (MABP, mmHg), **b.** Heart rate (HR, bpm); ***$p<0.05$** vs. baseline, # $p<0.05$: propranolol + DCA vs. control, $ $p<0.05$: propranolol + DCA vs. DCA. Means ± SE are presented

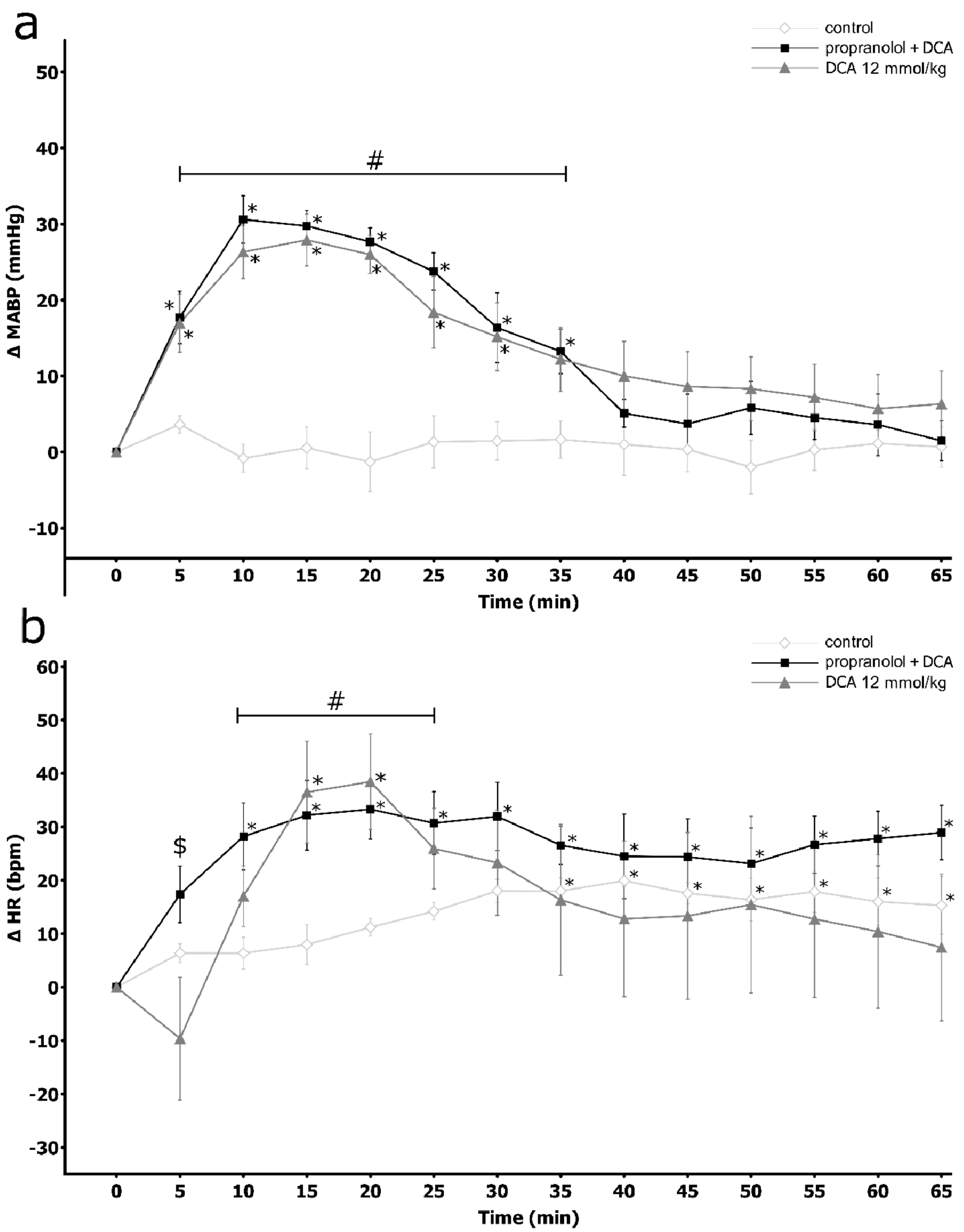

Figure S9

Changes in hemodynamic parameters in Sprague-Dawley rats after the intravenous administration (IV) of deoxycholic acid (DCA) at a dose of 12mmol/kg without pretreatment or after pretreatment with propranolol: DCA at a dose of 12 mmol/kg (propranolol+DCA) or the vehicle (control): **a.** ΔMABP (mmHg), **b.** ΔHR (bpm); ***$p<0.05$** vs. baseline, $^{\#}$ $p<0.05$: propranolol + DCA vs. control, $^{\$}$ $p<0.05$: propranolol + DCA vs. DCA. Means ± SE are presented

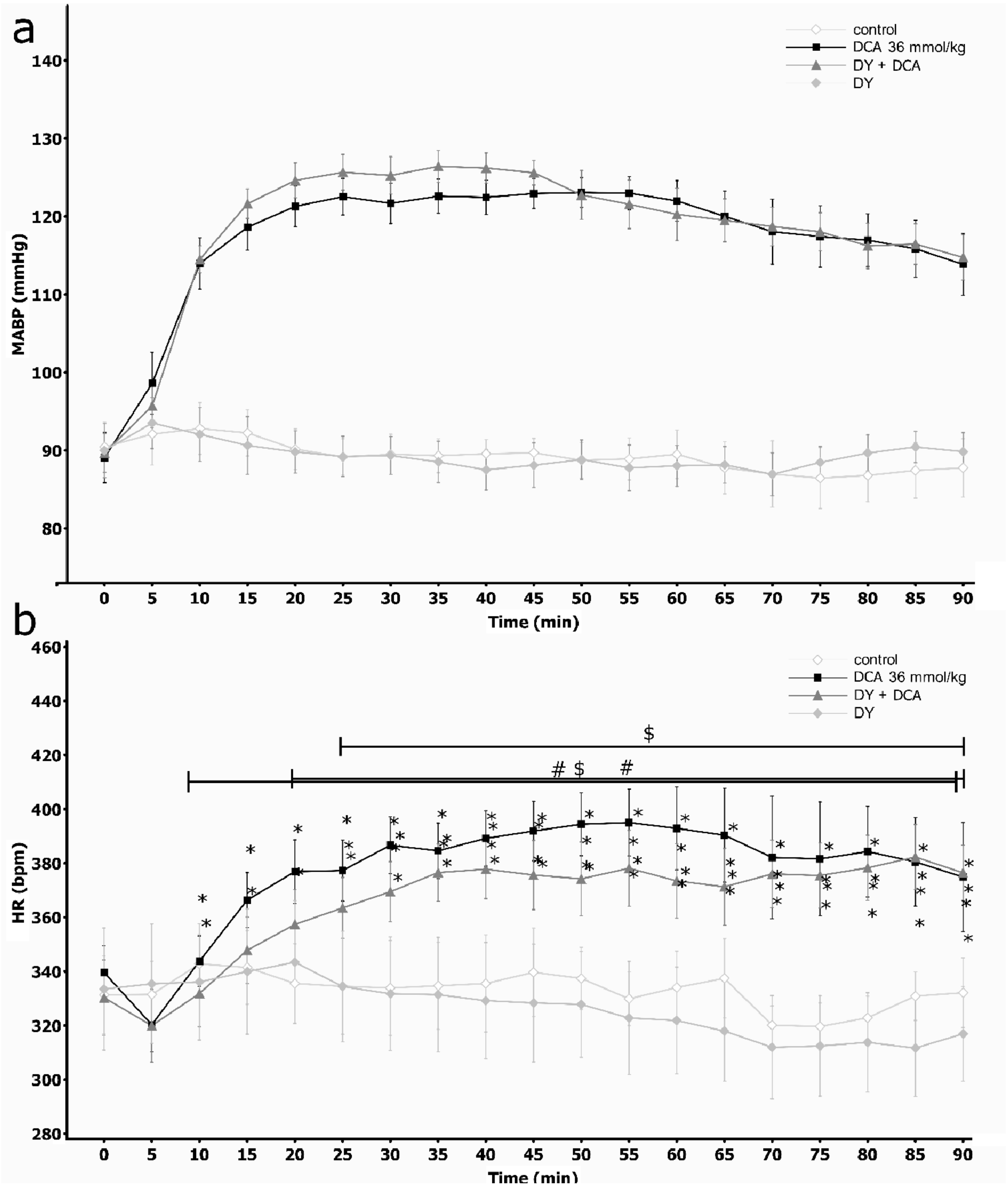

Figure S10

Hemodynamic parameters in Sprague-Dawley rats after the intravenous administration (IV) of deoxycholic acid (DCA) at a dose of 36 mmol/kg or the vehicle (control) without pretreatment or after pretreatment with DY 268: DCA at a dose of 36 mmol/kg (DY+DCA) or the vehicle (DY group): **a)** Mean arterial blood pressure (MABP, mmHg); **b)** Heart rate (HR, bpm); **$p<0.05$* vs. baseline, $^{\$}$ *$p<0.05$*: DY+DCA vs. DY, $^{\#}$ *$p<0.05$*: DCA vs. control. Means ± SE are presented

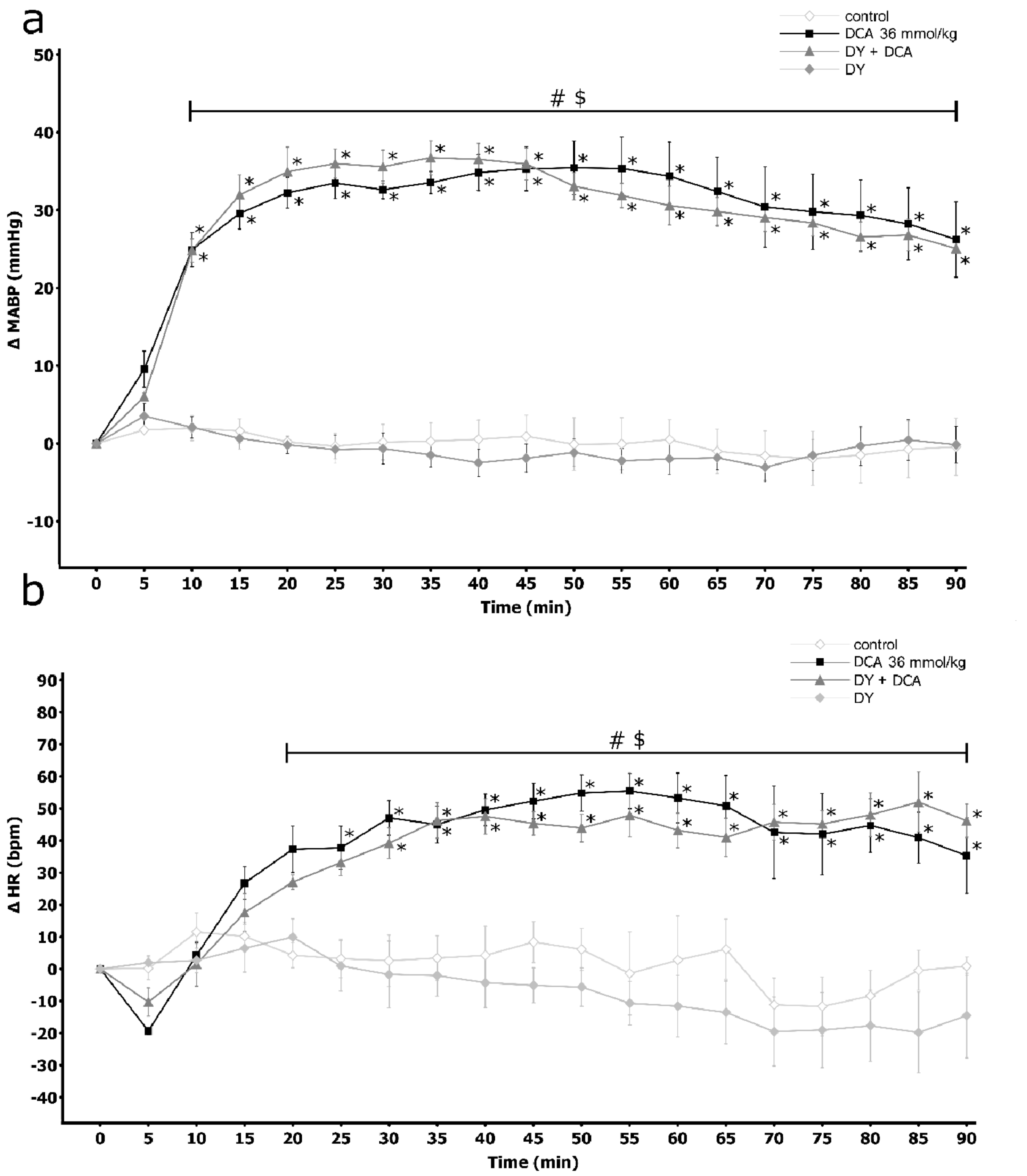

Figure S11

Changes in hemodynamic parameters in Sprague-Dawley rats after the intravenous administration (IV) of deoxycholic acid (DCA) at a dose of 36 mmol/kg or the vehicle (control) without pretreatment or after pretreatment with DY 268: DCA at a dose of 36 mmol/kg (DY+DCA) or the vehicle (DY group): **a)** ΔMABP (mmHg), **b)** ΔHR (bpm); ***$p<0.05$ vs. baseline, $^{\$}$ $p<0.05$: DY+DCA vs. DY, $^{\#}$ $p<0.05$: DCA vs. control. Means ± SE are presented

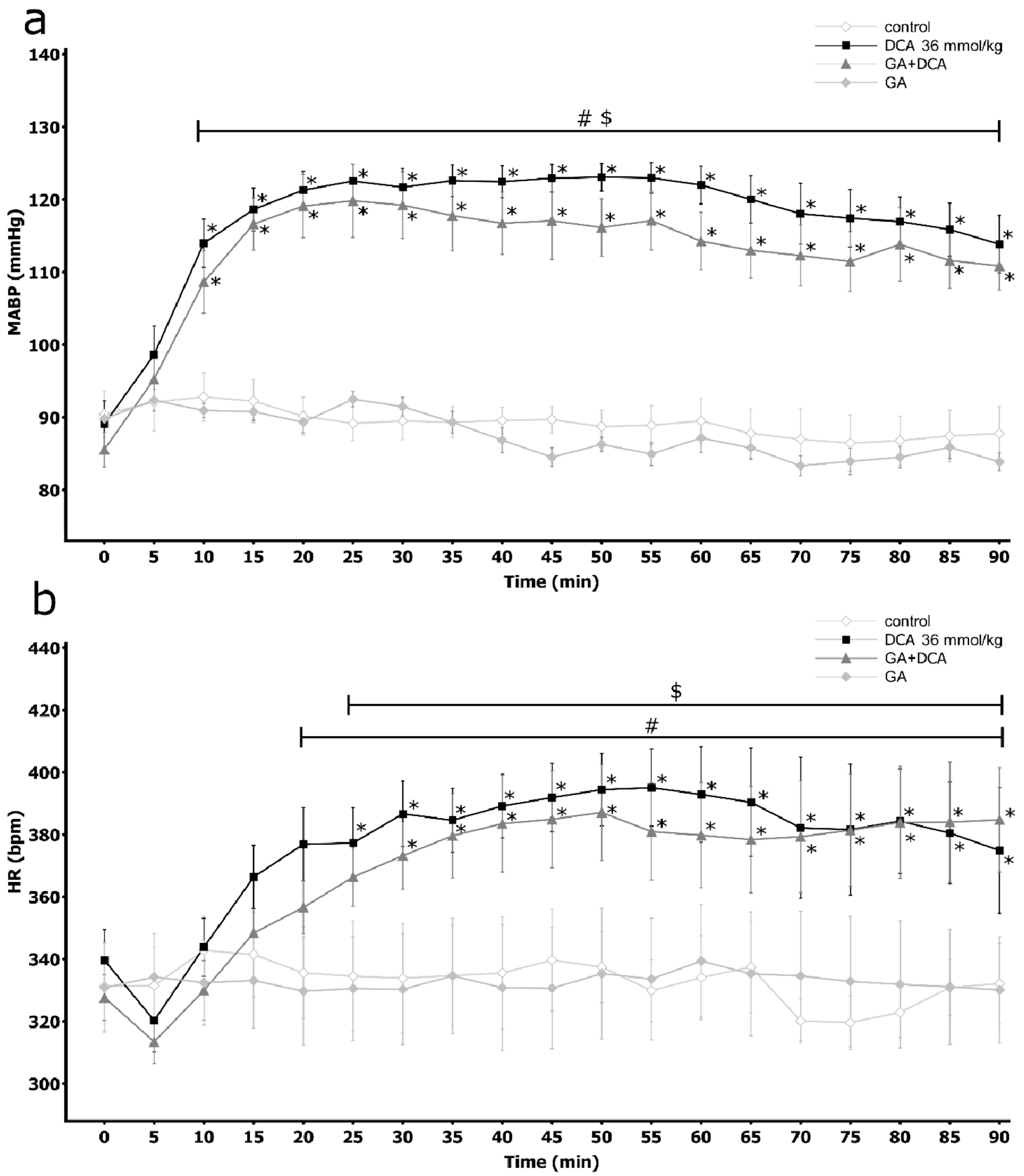

Figure S12

Hemodynamic parameters in Sprague-Dawley rats after the intravenous administration (IV) of deoxycholic acid (DCA) at a dose of 36 mmol/kg or the vehicle (control) without pretreatment or after pretreatment with glycyrrhetinic acid: DCA at a dose of 36 mmol/kg (GA+DCA) or the vehicle (GA group): **a)** Mean arterial blood pressure (MABP, mmHg), **b)** Heart rate (HR, bpm); *****$p<0.05$ vs. baseline, $^{\$}$ $p<0.05$: GA+DCA vs. GA, $^{\#}$ $p<0.05$: DCA vs. control. Means ± SE are presented

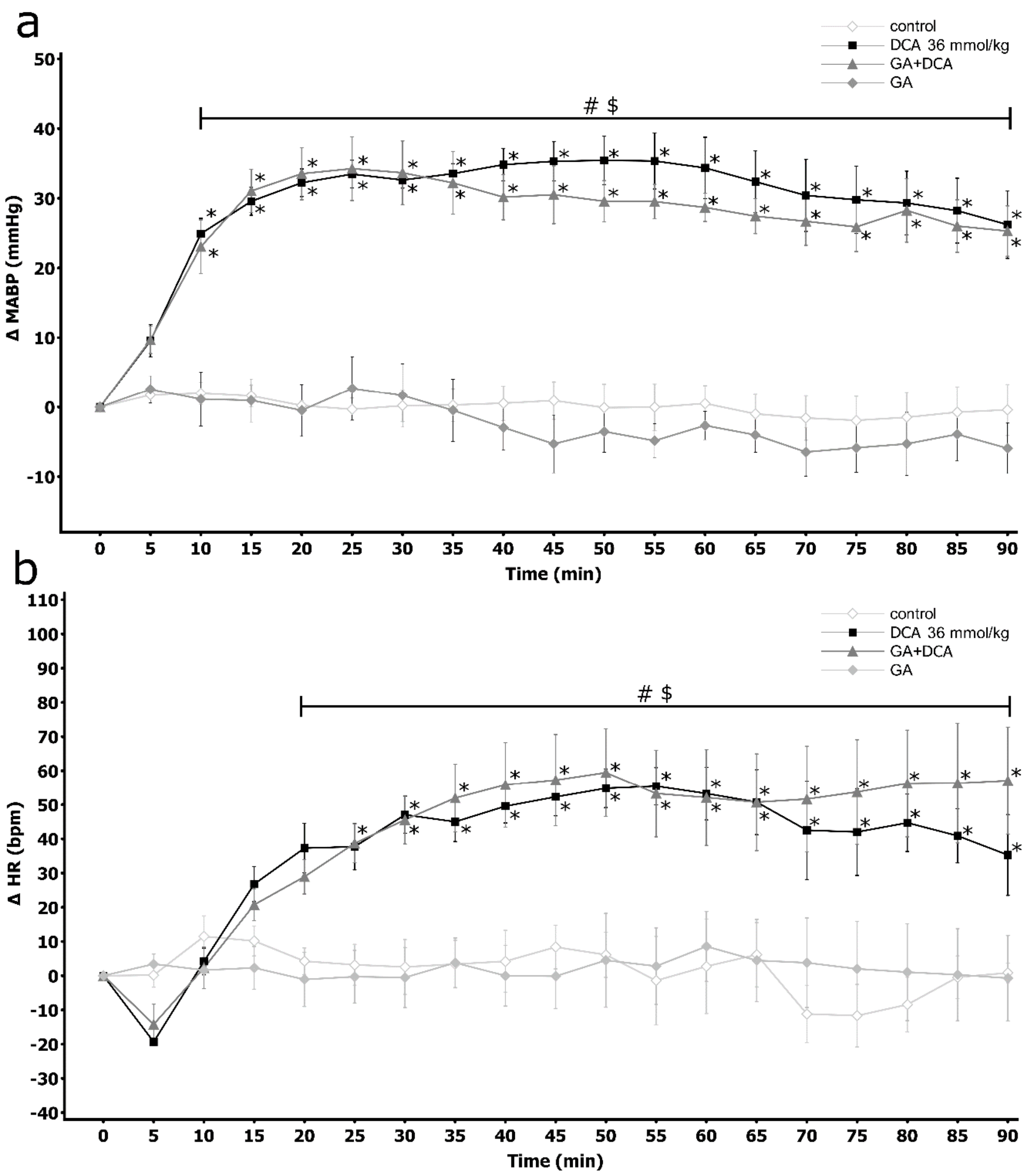

Figure S13

Changes in hemodynamic parameters in Sprague-Dawley rats after the intravenous administration (IV) of deoxycholic acid (DCA) at a dose of 36 mmol/kg or the vehicle (control) without pretreatment or after pretreatment with glycyrrhetinic acid: DCA at a dose of 36 mmol/kg (GA+DCA) or the vehicle (GA group): **a)** ΔMABP (mmHg), **b)** ΔHR (bpm); *****$p<0.05$ vs. baseline, $^{\$}$ $p<0.05$: GA+DCA vs. GA, $^{\#}$ $p<0.05$: DCA vs. control. Means ± SE are presented

INTRACEREBROVENTRICULAR ADMINISTRATION

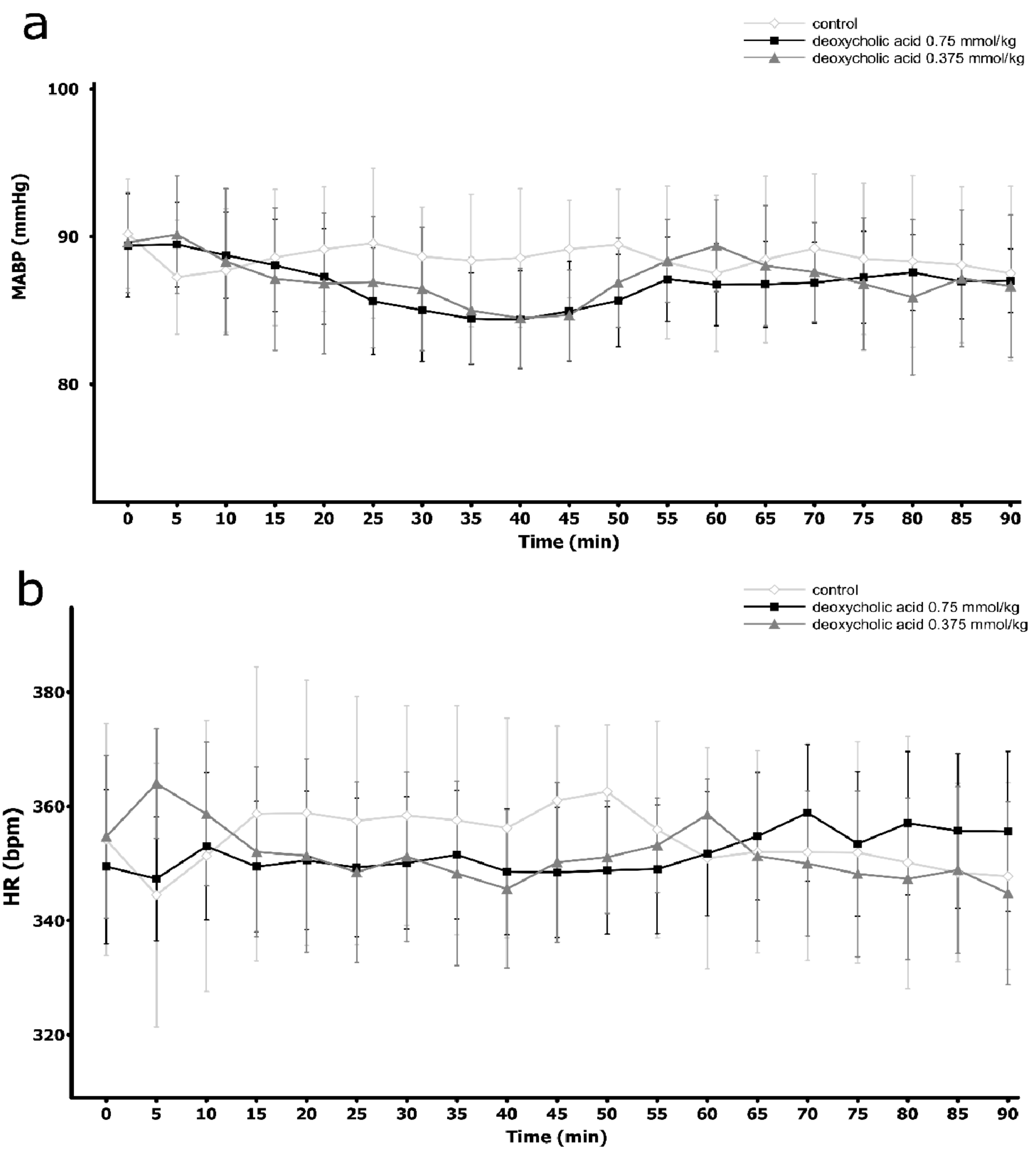

Figure S14

Hemodynamic parameters in Sprague-Dawley rats after the intracerebroventricular (ICV) administration of either the vehicle or deoxycholic acid (DCA) at a dose of 0.375 or 0.75 mmol/kg: **a)** Mean arterial blood pressure (MABP, mmHg), **b)** Heart rate (HR, bpm). No significant differences vs. baseline and between the groups. Means ± SE are presented

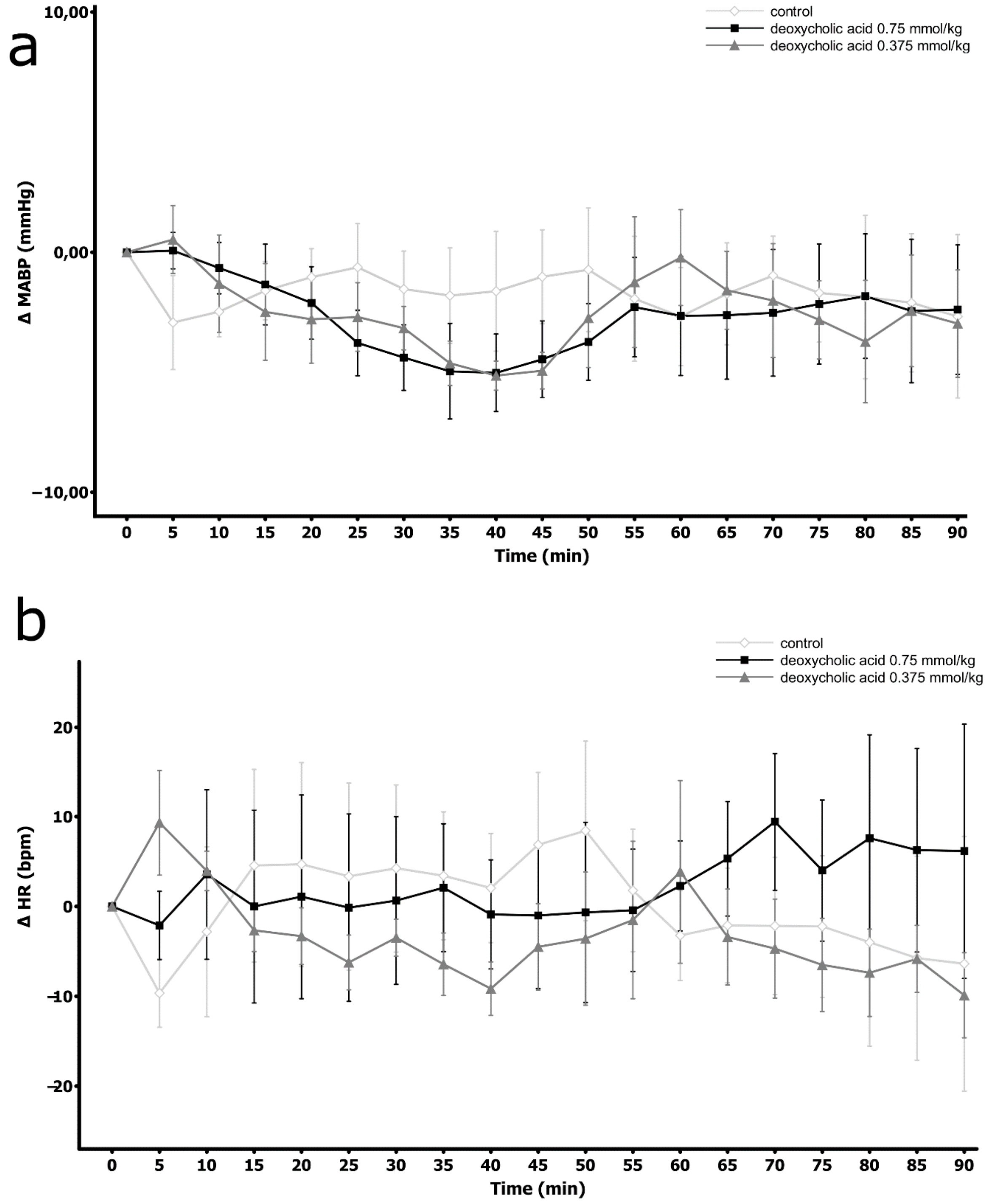

Figure S15

Changes in hemodynamic parameters in Sprague-Dawley rats after the intracerebroventricular (ICV) administration of either the vehicle or deoxycholic acid (DCA) at a dose of 0.375 or 0.75 mmol/kg: **a)** ΔMABP (mmHg), **b)** ΔHR (bpm). No significant differences vs. baseline and between the groups. Means ± SE are presented

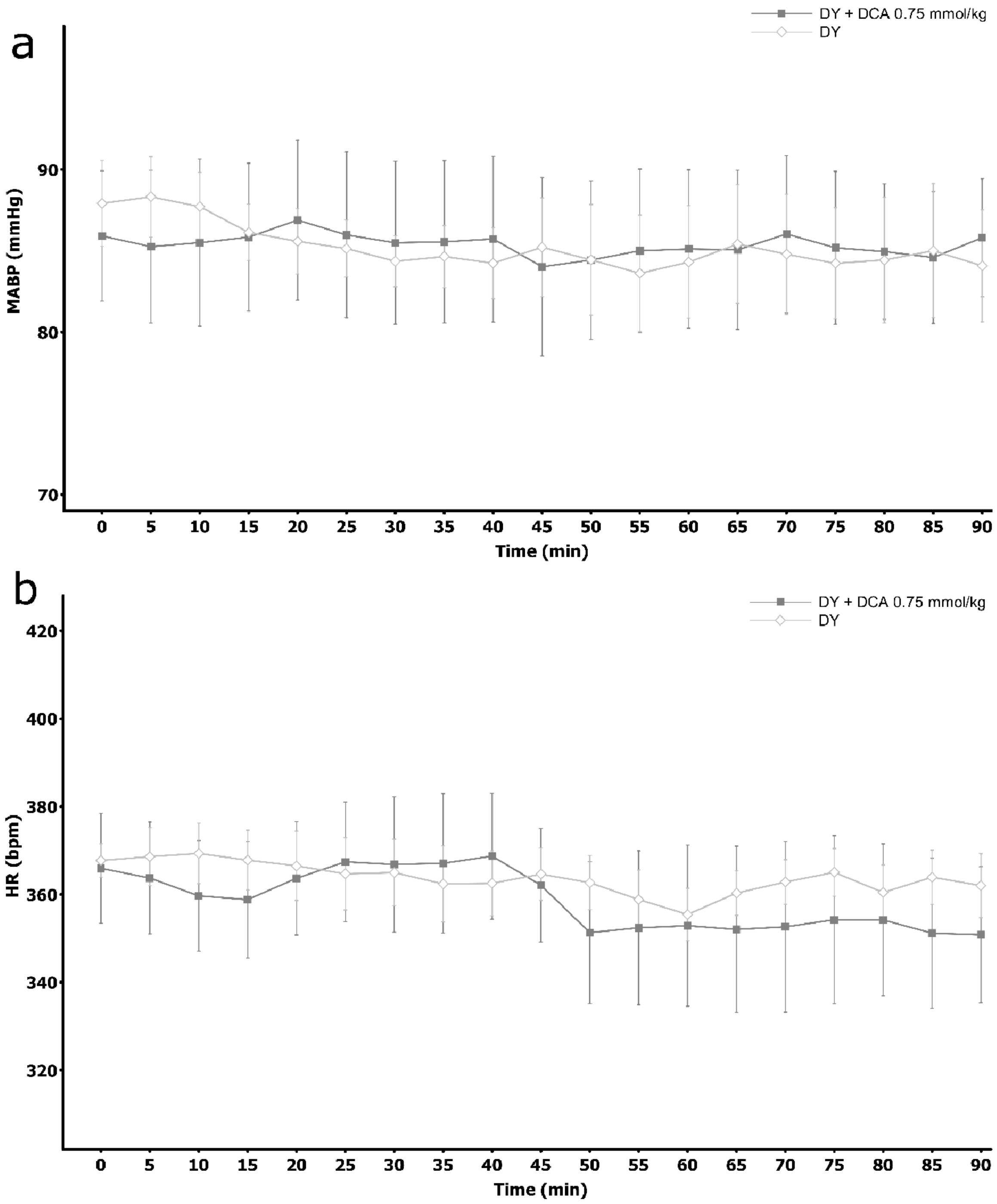

Figure S16

Hemodynamic parameters in Sprague-Dawley rats after the intracerebroventricular (ICV) administration of either the vehicle or deoxycholic acid (DCA) at a dose of 0.75 mmol/kg after pretreatment with DY 268: **a)** Mean arterial blood pressure (MABP, mmHg), **b)** Heart rate (HR, bpm). No significant differences vs. baseline and between the groups. Means ± SE are presented

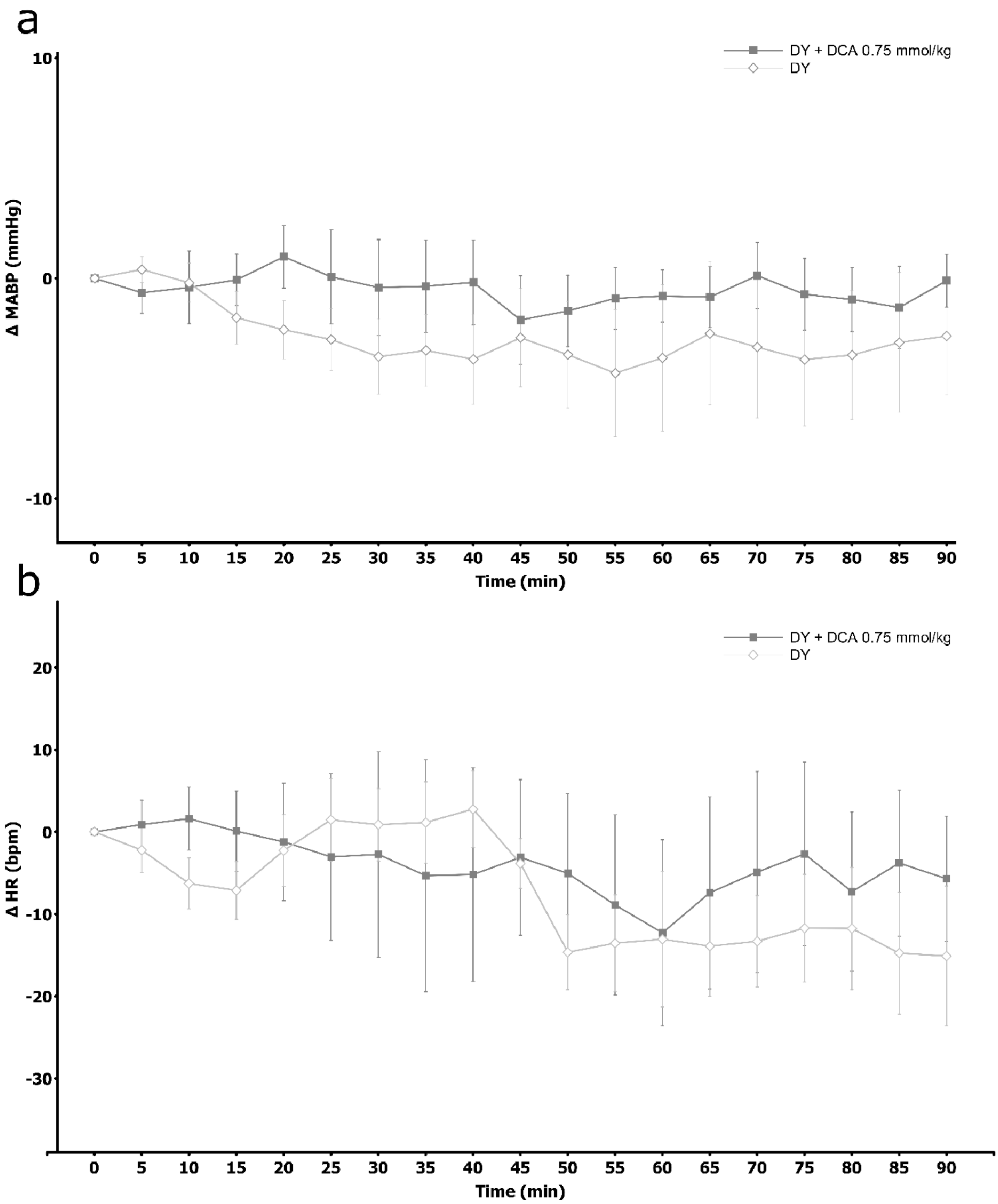

Figure S17

Changes in hemodynamic parameters in Sprague-Dawley rats after the intracerebroventricular (ICV) administration of either the vehicle or deoxycholic acid (DCA) at a dose of 0.75 mmol/kg after pretreatment with DY 268: **a)** ΔMABP (mmHg), **b)** ΔHR (bpm). No significant differences vs. baseline and between the groups. Means ± SE are presented

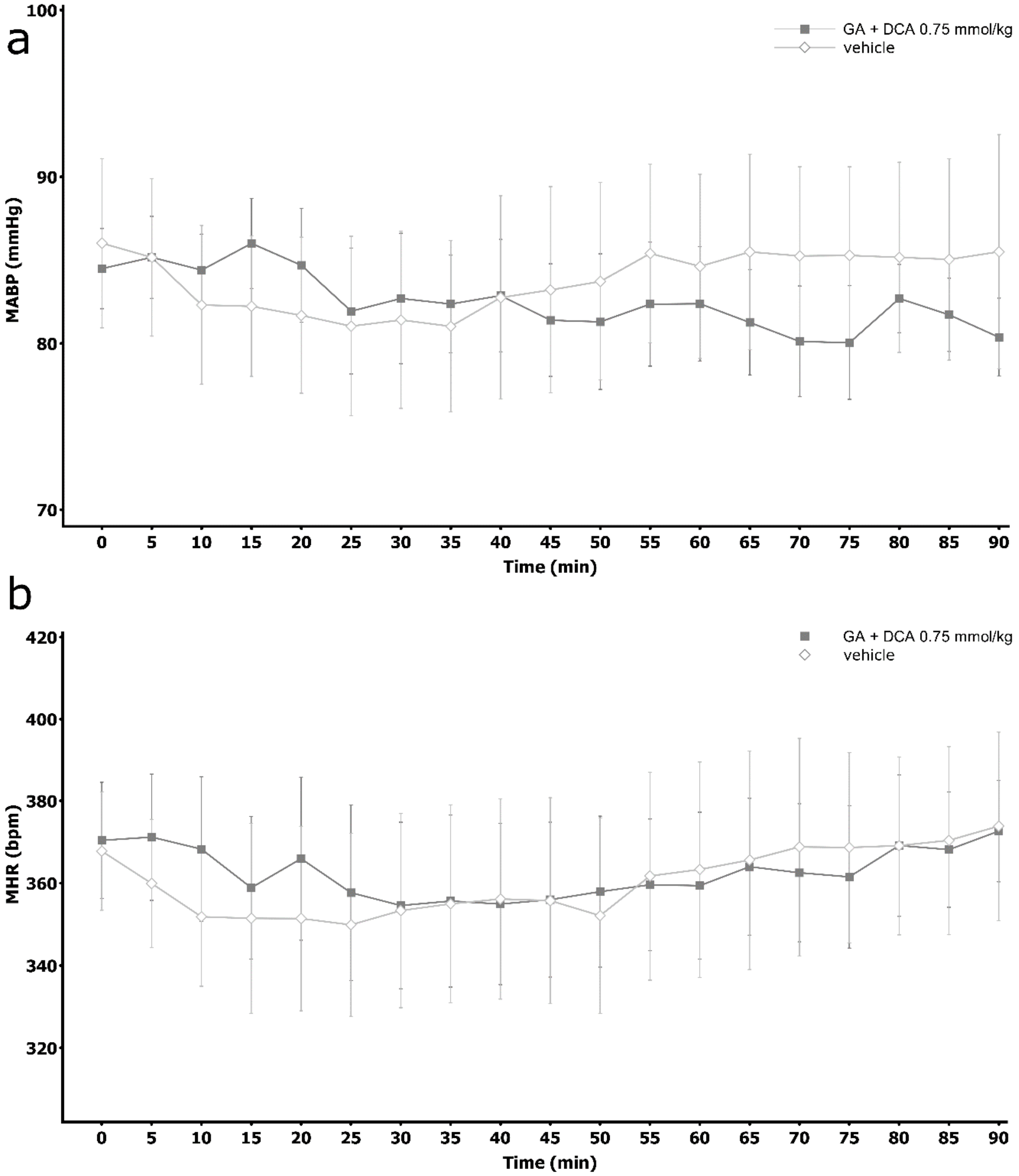

Figure S18

Hemodynamic parameters in Sprague-Dawley rats after the intracerebroventricular (ICV) administration of either the vehicle or deoxycholic acid (DCA) at a dose of 0.75 mmol/kg after pretreatment with glycyrrhetinic acid: **a)** Mean arterial blood pressure (MABP, mmHg), **b)** Heart rate (HR, bpm). No significant differences vs. baseline and between the groups. Means ± SE are presented

.

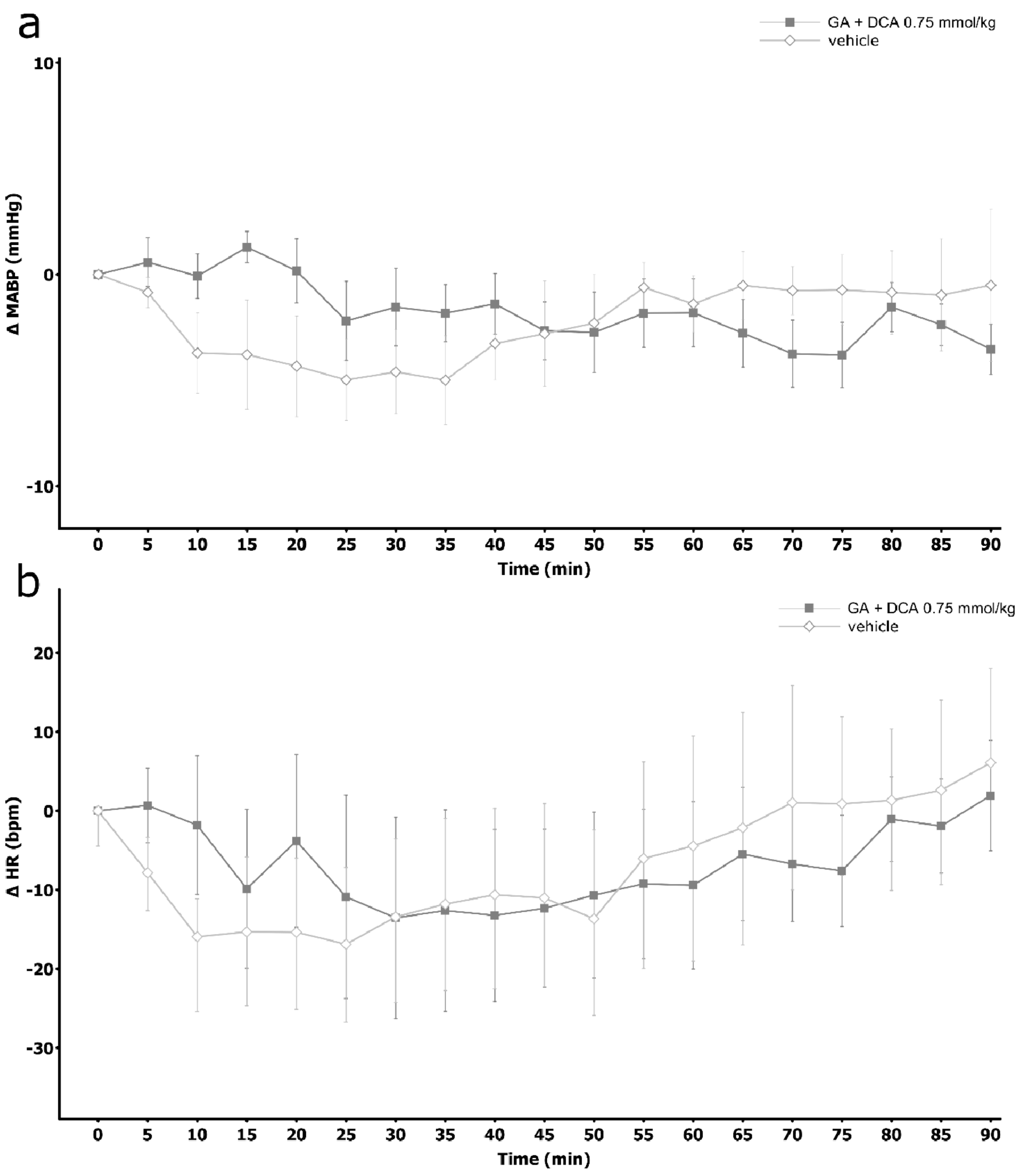

Figure S19

Changes in hemodynamic parameters in Sprague-Dawley rats after the intracerebroventricular (ICV) administration of either the vehicle or deoxycholic acid (DCA) at a dose of 0.75 mmol/kg after pretreatment with glycyrrhetinic acid: **a)** ΔMABP (mmHg), **b)** ΔHR (bpm). No significant differences vs. baseline and between the groups. Means ± SE are presented

SPECTROMETRY – DETAILED METHODS

Cholic and deoxycholic acid were determined using liquid chromatography coupled with tandem mass spectrometry (LC-MS/MS). Separation was achieved on a Kinetex C-18 column (100 mm × 4.6 mm, particle size 2.6 µm) supplied by Phenomenex (Torrance, CA, US) using Agilent 1260 Infinity (Agilent Technologies, Santa Clara, CA, USA) chromatograph connected to a hybrid triple quadrupole/linear ion trap mass spectrometer (QTRAP 4000; AB SCIEX, Framingham, MA, USA). Fifty µl of plasma was mixed with 10 µl of the internal standards solution (CA-D5 and DCA-D5, 10 µg mL^{-1}) and 200 µl of ice-cold acetonitrile. Next, the samples were vortexed for 5 min and centrifuged at 9,300 × *g* at 4°C for 5 min. The supernatant was transferred to a vial and analyzed. The curtain gas, ion source gas 1, ion source gas 2 and collision gas (all high purity nitrogen) were set at 241 kPa, 414 kPa, 275 kPa and "high" instrument units, respectively. The ion spray voltage and source temperature were set at -4500 V and 600°C, respectively. The chromatographic column was maintained at 40°C at a flow rate of 0.5 mL min^{-1}. The mobile phases consisted of a water solution of 0.2% formic acid as eluent A and acetonitrile with 0.2% formic acid as eluent B. The gradient (%B) was as follows: 0 min 80%; 0.5 min 80%; 4 min 5%; 9.5 min 5%. The volume of injection was 10 µL. The target compounds were analyzed in multiple reaction monitoring mode. The transitions used for quantitation were *m/z* 407>343 and *m/z* 391>345 and *m/z* 412>348 and *m/z* 396>350 for CA, DCA and CA-D5 and DCA-D5, respectively. The compound parameters, viz. declustering potential (DP), collision energy (CE), entrance potential (EP) and collision cell exit potential (CXP) were -150 V, -44 V, -10 V, -9 V for CA, -155 V, -46 V, -10 V, -17 V for DCA, -145 V, -44 V, -10 V, -15 V for CA-D5 and -130 V, -48 V, -10 V, -9 V for DCA-D5.

EX VIVO REACTIVITY STUDIES - DETAILED METHODS

Rats (n = 6) were anesthetized with an intraperitoneal injection of a 15% urethane solution (1.5 g/kg). The mesenteric artery was dissected and placed in a petri dish filled with cold (4 °C, pH = 7.4) physiological saline buffered with MOPS (3-(N-morpholino) propanesulfonic acid) (MOPS-PSS) containing: 3.0 mM MOPS, 44.0 mM NaCl, 3.0 mM KCl, 2.5 mM $CaCl_2$, 1.5 mM $MgSO_4$, 1.21 mM NaH_2PO_4, 0.02 mM EDTA, 2.0 mM sodium pyruvate, 5.0 mM glucose, and 1% dialyzed bovine serum albumin (BSA). The MAs were cleaned of surrounding tissues and transferred to an organ chamber, which was placed on the stage of the inverted microscope (CKX41, Olympus, Germany) equipped with a video camera and a monitor. The transmural pressure was set at 50 mmHg. The experiments were performed without intraluminal flow. After 60 min equilibration at 37°C, the arteries were preconstricted with phenylephrine (PE, 1 µM). After the contraction reached a steady state, acetylcholine (ACh, 1 µM) was added to MOPS-PSS to assess the integrity of the endothelium. Deoxycholic acid was administered extravascularly at increasing concentrations from 0.1 µM to 500 µM. The starting concentration (0.1 µM) of the deoxycholic acid was lower than its physiological concentration measured in the plasma of the rats to make sure that the sub-physiological dose did not cause a vascular effect. The highest dose of deoxycholic acid (500 µM) was the dose that produced maximal dilation of mesenteric arteries. The effects of each concentration of the tested substances on the inner diameter of MAs were assessed 15 min after the substances were administered. At the end of each experiment, the MOPS-PSS bath solution was replaced with Ca^{2+}-free PSS (PSS containing 3 mM EGTA) to achieve maximal dilation of the vessel. All values are expressed as means ± SE. Vasodilatation, as a percent of the maximal diameter, was calculated based on a formula $(D_{active} - D_{baseline}) / (D_{passive} - D_{baseline}) \times 100\%$, where D_{active} is the measured diameter for a given dose of the tested compound, $D_{baseline}$ is the baseline diameter measured before administration of the drug, and $D_{passive}$ is the maximal passive diameter.
